# Efficient One-Pot Solvothermal Synthesis and Characterization of Zirconia Nanoparticle-Decorated Reduced Graphene Oxide Nanocomposites: Evaluation of Their Enhanced Anticancer Activity toward Human Cancer Cell Lines

**DOI:** 10.1021/acsomega.2c06822

**Published:** 2023-01-03

**Authors:** Nalinee Kanth Kadiyala, Badal Kumar Mandal, L. Vinod Kumar Reddy, Crispin H. W. Barnes, Luis De Los Santos Valladares, Dwaipayan Sen

**Affiliations:** † Trace Elements Speciation Research Laboratory, Department of Chemistry, School of Advanced Sciences, Vellore Institute of Technology (VIT), Vellore 632014, India; ‡ Cellular and Molecular Therapeutics Laboratory, Centre for Biomaterials, Cellular and Molecular Theranostics, Vellore Institute of Technology (VIT), Vellore 632014, India; § Cavendish Laboratory, Department of Physics, University of Cambridge, Cambridge CB3 0HE, United Kingdom; ∥ Faculty of Physics and Technology, L.N. Gumilyov Euroasian National University, Nur-Sultan 010000, Kazakhstan; ⊥ Laboratorio de Cerámicos y Nanomateriales, Facultad de Ciencias Físicas, Universidad Nacional Mayor de San Marcos, Ap. Postal 14-0149, Lima 14-0149, Peru

## Abstract

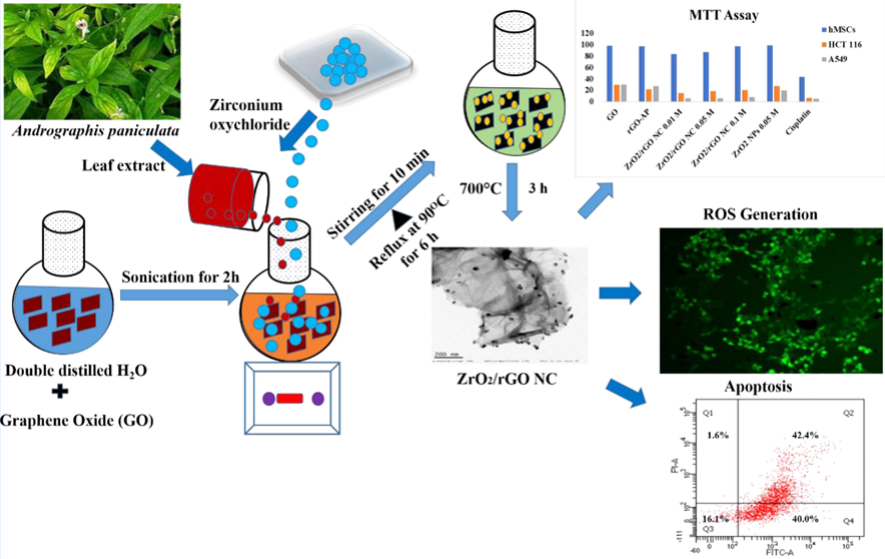

This study mainly deals with an effective one-pot solvothermal synthetic pathway for the preparation of uniformly dispersed zirconium oxide nanoparticles on the flattened rough surface of reduced graphene oxide (ZrO_2_/rGO NCs) using the aqueous leaf extract of *Andrographis paniculata*. After obtaining detailed information on the preparation and characterization, the anticancer activity of the synthesized ZrO_2_/rGO nanocrystals (NCs) was evaluated on two human cancer cell lines (A549 and HCT116) along with one normal human cell line (hMSC). The 3-[4,5-dimethylthiazole-2-yl]-2,5-diphenyltetrazolium bromide assays revealed that ZrO_2_/rGO NCs exhibited a dose-dependent cytotoxicity pattern. The cell viability (%) drastically decreases up to 96–98% after exposure to an optimal concentration of 10 ppm nanocomposites. Analysis of both the reactive oxygen species generation and the Annexin V-FTIC staining assays reveal that ZrO_2_/rGO NCs have the ability to induce apoptosis in A549 and HCT116 cell lines. Thus, the green synthesis of ZrO_2_/rGO NCs shows potential in developing efficient therapeutic agents for cancer therapy.

## 1 Introduction

Cancer is described as the uncontrolled proliferation of cells in an abnormal direction leading to unique diseases, and in some cases, it also metastasizes to distinctive sites. In the current decade, cancer has been considered as the most serious health problem and is the second major reason for death in the world. The death rate is also soaring rapidly with increasing population and aging and is associated with socioeconomic development.^1^ Based on the type of cancer, the National Cancer Institute (NIH) of the USA has summarized existing treatments such as radiation therapy, targeted therapy, immunotherapy, hormone therapy, chemotherapy, precision medicine, stem cell therapy, and surgery.^2^ However, these therapies or curative methods have manifold side effects that can alter the mortality rate. In 2015, the World Health Organization (WHO) stated that cancer is a prominent reason for morbidity and mortality globally, affecting more than 14 million people yearly. Cancer cells are well-known to develop resistance against existing curative methods, which demands the development of new directions to deal with cancer.^3^ Apoptosis is a very well-regulated method of predetermined cell death in which cells go through a closely regulated program such as nuclear collapse, cellular shrinkage, fragmentation of nuclear material, and formation of apoptotic bodies that diminish inflammation and cause damage to surrounding cells. In general, uninhibited proliferation and loss of apoptotic control are the key factors for tumor formation. Thus, compounds that inhibit tumor cell proliferation by inducing apoptosis have recently attracted high interest in cancer treatment research.^4^

Recent studies report that graphene and related components such as graphene oxide (GO), reduced graphene oxide (rGO), and reduced graphene oxide nanocomposites (rGO/NCS) are good candidates for different biomedical applications.^5,6^ Graphene is composed of an sp^2^-hybridized two-dimensional (2D) carbon structure like a 2D honeycomb lattice with exceptional and remarkable physiochemical properties such as high electrical, thermal, mechanical, and optical properties along with a high surface area, cytocompatibility, and degradability.^7−14^ In addition to these properties, graphene has also been reported to serve as an anti-cancer drug delivery agent by releasing drugs at targeted cancer cells.^15,16^ Despite the fact that reports on biomedical activities of graphene and graphene oxide are increasing tremendously, relatively very little is known about their intrinsic toxicity or biological interface. Besides, the major challenge is to regulate the biological interaction of reduced graphene oxide (rGO) materials for their safe and efficient utilization in a cell system interface.^17^ Consequently, the initial approach to characterize the cytotoxicity of any nanocomposite material is currently an *in vitro* assay.

Numerous studies have been performed to determine the graphene toxicity levels in various cell types such as MCF-7, human breast cancer, HeLa, ovarian cancer, primary mouse embryonic fibroblast, SKBR3, and phenochromocytoma-derived PC12, NIH3T3, and HepG2 cell lines using *in vitro* assays.^18^ The usual mechanisms of rGO-based cell toxicity assays suggest damage to the plasma membrane, diminishing of mitochondrial activity, DNA impairment, oxidative stress induction, and ultimately apoptotic or necrotic cell death.^19−23^ However, there are some conflicting reports, particularly with GO, due to the discrepancies in the intrinsic properties of the tested nanomaterials and cell line sensitivity against nanomaterials while performing the assay. Overall, the biocompatibility and cytotoxicity of rGO NCs depend on the type of reducing agent used and their obtained particle size.^24^

Metal oxide nanostructures have shown promising electrical and optical properties due to the phonon and electron confinement, high catalytic activity, high surface-to-volume ratio, revised surface work function, and strong adsorption ability.^25^ In the case of ZrO_2_ nanoparticles (NPs), their low toxicity and high chemical inertness make them environmentally friendly materials as well as prospective biological agents.^26^ The promising properties of ZrO_2_ NPs, i.e., biocompatibility, thermal stability, and low-cost production, make them superior materials in many catalytic and sensing applications^27−34^ as well as other biological systems such as plants and microorganisms. Interestingly, sulfated ZrO_2_ NPs were evaluated for their toxicity effect against liver cancer and colon cancer cells with significant results.^35^ Also, the impact of ZrO_2_ NPs on the *Pseudomonas putida* bacterial strain growth was reported.^36,37^ Mogha et al. reported the biocompatible ZrO_2_/rGO nanocomposite and utilized it as a biosensor for the chlorpyrifos molecule.^38^ Sai Saraswathi et al. investigated the cytotoxic activity of ZrO_2_ NPs toward MCF-7 cell lines.^39^ Similarly, Balaji et al. reported the cytotoxic activity of ZrO_2_ NPs toward HCT-116 and A549 cell lines and proposed a mechanism of activity using the DCFH2-DA dye assay. They also confirmed that while the generated reactive oxygen species (ROS) were responsible for the cytotoxicity of ZrO_2_ NPs toward cancer cell lines,^40^ the molecular mechanism of ROS generation/oxidative stress and nanoparticle-mediated cell death were well described.^40,66,67^ Here, graphene behaved as an electron acceptor to prevent electron–hole pair recombination, which further induced ROS generation.^41^ This basic mechanism is the key factor in nanomaterial-mediated cytotoxicity, DNA damage, apoptosis, and cancer.^65^

Several methods are available to prepare ZrO_2_/rGO NCs, such as hydrothermal methods^38^ electrochemical synthesis,^42^ and solution-based methods.^43^ However, the reported methods demand high temperatures, long reaction times, and high pressure and release undesired byproducts. Moreover, the majority of these methods report the separate syntheses of rGO and ZrO_2_ NPs, followed by combining them in a solution, which is a limitation for large-scale synthesis procedures. To overcome this issue, an alternative method has been established in this study to synthesize rGO/ZrO_2_ NCs in one pot, which is cost-effective and simple. To avoid the use of hazardous agents, several biocompatible and environmentally friendly reducing/capping agents such as humanin, melatonin, plant extracts, and vitamins have been introduced for the effective reduction of GO to rGO.^44−48^

Herein, we utilize the aqueous leaf extract of *Andrographis paniculata* as a unique and low-cost capping and reducing agent for the synthesis of evenly decorated ZrO_2_ NPs on the rGO surface. *A. paniculata* belongs to the *Acanthaceae* family, which is commonly known as Nilavembu in India and found in many parts of India, Southeast Asia, Sri Lanka, and China. This herb exhibits a wide range of pharmacological activities such as anti-inflammatory, antimicrobial, hypoglycemic, antioxidant, cardioprotective, hepatoprotective, anticancer, and antiallergic properties.^49,50^ Furthermore, this plant contains many active phytochemicals such as flavonoids, steroids, terpenoids, glycosides, alkaloids, tannins, gums, saponins, phenolic compounds, and stigma steroids,^51^ which promote the facile reduction of GO and zirconium ions (Zr^4+^ ions) spontaneously, leading to the formation of ZrO_2_/rGO NCs. To the best of our knowledge, this is the first report on the biogenic solvothermal synthesis of ZrO_2_/rGO NCs using the leaf extract of *A. paniculata* and the evaluation of their anticancer activity. Also, there are no reports on the mechanism of apoptosis in cancer cells induced by the dissolution of ZrO_2_ NPs from the rGO surface. The current study dealt with the synthesis, characterization, and cytotoxic activity study of ZrO_2_/rGO NCs against human cancer cells, i.e., HCT116 (colorectal) and A549 (lung), and mesenchymal stem cells that were derived from the human umbilical cord blood (hMSCs). The synthesized ZrO_2_/rGO NCs were used as target materials to condense the cancer cells. The decline in the cell viability percentage and apoptosis induced by these nanocomposites were systematically analyzed by the 3-(4,5-dimethylthiazol-2-yl)-2,5-diphenyltetrazolium bromide (MTT) assay and flow cytometry analysis, respectively.

## 2 Materials and Methods

### 2.1 Materials

Graphite powder (100 mesh, 99.9% pure), potassium permanganate (KMnO_4_, >99%), sulfuric acid (H_2_SO_4_, 98%), hydrogen chloride (HCl, 36%), hydrogen peroxide (H_2_O_2_, 30%), and zirconium oxychloride (ZrOCl_2_·8H_2_O, >99%) were obtained from Sigma Aldrich Bangalore, India. Sodium nitrate (NaNO_3_, AR grade, Qualigens, India), Dulbecco’s modified Eagle’s medium (DMEM, HI Media), fetal bovine serum (FBS, HI Media), α-minimum essential medium (α-MEM, Gibco/Life Technologies), 3-(4,5-dimethylthiazol-2-yl)-2,5-diphenyltetrazolium bromide (MTT, HI Media), 1% pen/strep (HI Media), human umbilical cord blood-derived mesenchymal stem cells (hMSCs) (PromoCell, Germany), human colon (HCT116) and lung (A549) carcinoma cell lines (National Sciences for Cell Sciences, NCCS, Pune, India, American Type Culture Collection, ATCC), Drug Cisplat (cisplatin injection I.P. 50 mg/50 mL, Zydus Oncosciences), and double-distilled water were used throughout the experiment.

### 2.2 Preparation of the *A. paniculata* Leaf Extract

Nilavembu plant leaves (*A. paniculata*) were collected from the surroundings of Vellore Institute of Technology (Tamil Nadu) and thoroughly rinsed using deionized water to clean the adsorbed dirt and air dried for 2 h. The dried leaves were further chopped into tiny pieces and shade dried for 7 days at room temperature (RT) (25 °C). Then, dried leaves were powdered and sieved to obtain a fine powder. About 100 mL of double-distilled water was added to 5 g of *A. paniculata* leaf powder and heated at 90 °C/10 min. The aqueous solution changed to a thick blackish-brown color, and the resultant colored extract was allowed to reach room temperature, followed by filtration using the Whatman No. 1 filter paper. The final extract was stored at 4 °C for further studies.

### 2.3 Synthesis of ZrO_2_/rGO NC and rGO-AP

A modified Hummer’s method was utilized to synthesize GO.^52,53^ A one-pot solvothermal green synthetic route was employed to synthesize the GO nanocomposite using the *A. paniculata* leaf extract. The starting GO material was added to water (1 mg mL^–1^) and sonicated for 2 h to obtain a homogeneous dispersion. Zirconium oxychloride (ZrOCl_2_·8H_2_O) was added to the dispersed GO and stirred for 10 min. After the vigorous dissolution of the GO–zirconium oxychloride mixture, the pH of the final solution was adjusted to 9.0 using NaOH (1M) solution, and the leaf extract of *A. paniculata* (10 mL) was added dropwise using a peristaltic pump. After the complete addition of the leaf extract, the final solution was refluxed at 90 °C for 6 h. Then, the reactant solution was allowed to reach room temperature until its color changed to gray. It was then centrifuged several times and washed thoroughly with deionized water to remove the unreacted materials and finally dried at 80 °C overnight.

Three different doping concentrations of zirconium oxychloride (0.01, 0.05, and 0.1M) were utilized to fabricate the different rGO nanocomposites following calcination at 700 °C for 3 h at a constant heating rate of 1 °C min^–1^ under ambient conditions. The obtained products were labeled ZrO_2_/rGO (0.01M), ZrO_2_/rGO (0.05M), ZrO_2_/rGO (0.1M) NCs, and ZrO_2_ NPs (0.05M). The ZrO_2_ NPs were synthesized from the zirconium precursor under similar experimental conditions without GO. The starting material GO was further reduced to rGO using the leaf extract under similar experimental conditions and labeled rGO-AP. All of the synthesized materials were characterized using different techniques to confirm the formation of the synthesized products.

### 2.4 Material Characterization

Crystallographic information was obtained using X-ray diffraction (XRD) patterns (Brucker D8 Advance diffractometer). Fourier transform infrared (FTIR) spectroscopy was carried out in attenuated total reflectance (ATR) mode (JASCO ATR-FTIR 4100). Scanning electron microscopy (SEM) and energy-dispersive X-ray (EDX) spectroscopy analyses were performed using a Carl Zeiss SEM instrument. TEM images and the selected area electron diffraction (SAED) patterns were recorded by high-resolution TEM (HR-TEM) (JEOL JEM 2100). X-ray photoelectron spectroscopy (XPS) measurements were performed using an ESCA-3000 (VG Scientific, U.K.), and the C 1s and O 1s spectra were successfully deconvoluted using XPS Peak 4.1 software. ζ-Potential and dynamic light scattering (DLS) measurements were performed using a Horiba Scientific Nanoparticci (SZ-100) instrument. Sample preparation for instrumental analyses was carried out by effective dispersion (0.5 mg mL^–1^) of the respective compound in an aqueous solution. A fluorescence microscope (Model No FM-3000, Weswox, Ambala) was used to record the fluorescence microscopic images of all cell lines to confirm the generation of ROS after exposure to the respective synthesized nanomaterials.

### 2.5 Cell Culture and Exposure to ZrO_2_/rGO Nanocomposites

The human colorectal carcinoma cell line HCT116 and adenocarcinomic alveolar basal epithelial cells A549 were obtained from the National Sciences for Cell Sciences (NCCS, Pune, India), American Type Culture Collection (ATCC), and one normal cell line of human umbilical cord blood-derived mesenchymal stem cells (hMSCs) was procured from PromoCell (Germany) to govern the cell viability upon exposure to ZrO_2_/rGO NCs.

Cell lines were cultured in DMEM/α-MEM medium, which was supplemented with fetal bovine serum (10%) and penicillin/streptomycin (1%). The prepared cultures were maintained in an incubator at 37 °C under a 5% CO_2_ and 95% humidified atmosphere. The medium of every culture was switched once every 3 days. All of the experiments were performed with cells of passage numbers 2 and 5. Cells were harvested at 85% confluence and transferred into subcultured flasks of dimensions 75 cm^2^ and six-well plates or 96-well plates based on the experiment using 0.25% trypsin. Cells were allowed to attach to the surface for 24 h before treatment. ZrO_2_/rGO NCs were suspended in the cell culture medium and diluted to appropriate concentrations (1, 2, 4, 6, 8, and 10 ppm). The dilutions of ZrO_2_/rGO NCs were then subjected to sonication using a bath sonicator at room temperature for 10 min at 40 W to avoid agglomeration of the nanocomposites before cell exposure. Following treatment, cells were harvested to determine the cytotoxicity, generation of reactive oxygen species (ROS), and apoptosis markers. Control samples were also investigated in the absence of ZrO_2_/rGO NCs under similar experimental conditions.

### 2.6 Cell Viability and MTT Assay

The 3-(4,5-dimethylthiazole-2-yl)-2,5-diphenyltetrazolium bromide (MTT) assay conditions with a few modifications were employed to determine the viability of A549, HCT116, and hMSC cell lines.^55^ In brief, 1 × 10^4^ cells/well were seeded into 96-well plates and subjected to GO, rGO-AP, ZrO_2_/rGO (0.01M), ZrO_2_/rGO (0.05M), ZrO_2_/rGO (0.1M) NCs, and ZrO_2_ NPs (0.05M) at 0, 1, 2, 4, 6, 8, and 10 ppm concentrations for 24 h. After exposure, the culture medium was discarded from every well to avoid interference of ZrO_2_/rGO NCs and substituted with fresh medium comprising MTT solution (0.5 mg mL^–1^) in an appropriate amount equivalent to 10% culture volume and further incubated at 37 °C for 3 h till the development of a purple-colored formazan product. The subsequent formazan crystalline product was dissolved in dimethyl sulfoxide (DMSO). The colorimetric reaction was monitored using a multiwell plate reader, and the absorbance changes were recorded at 595 nm. Finally, the cell viability results (%) were articulated with respect to the control results.

### 2.7 Reactive Oxygen Species (ROS) Detection

The generated intracellular ROS was assessed corresponding to the modified method of Wilson et al. utilizing 2,7-dichlorofluorescein diacetate (DCFH-DA).^55^ The nonfluorescent compound DCFH-DA undergoes intracellular deacetylation and ROS-mediated oxidation and is finally converted to dichlorofluorescein (DCF), which is highly fluorescent in nature. In short, 10 mM DCFH-DA stock solution (in methanol) was diluted in the culture medium in the absence of serum or any other additive to generate a 100 μM working solution. A549, HCT116, and hMSC cell lines were treated with GO, rGO-AP, ZrO_2_/rGO (0.01M), ZrO_2_/rGO (0.05M), ZrO_2_/rGO (0.1M) NCs, and ZrO_2_ NPs (0.05M) at a concentration of 10 ppm for 24 h. After the exposure, cells were rinsed twice with Hank’s balanced salt solution (HBSS) and then incubated in 1 mL of DCFH-DA (working solution) at 37 °C for 30 min. The cells were lysed in an alkaline medium solution and centrifuged for 10 min at 2500 rpm. Then, 200 μL of the supernatant solution was transferred to a 96-well plate, and the fluorescence microscopic images were recorded to evaluate ROS generation. The mean fluorescence intensity (MFI) of the ZrO_2_/rGO NC-mediated ROS was quantified through NIH ImageJ software program. The recorded MFI data were stated as the dichlorofluorescein (DCF) relative fluorescence intensity.

### 2.8 Evaluation of the Mode of Cell Death (Apoptosis/Necrosis) in Exposed Cells

An apoptosis kit with Annexin V Alexa Fluor 488 and propidium iodide (PI) (Thermofisher) was utilized to detect apoptotic and necrotic cells after being subjected to GO, rGO-AP, ZrO_2_/rGO (0.01M), ZrO_2_/rGO (0.05M), ZrO_2_/rGO (0.1M) NCs, and ZrO_2_ NP (0.05M). The kit manual was stringently followed for all experiments. This assay was performed according to the procedure developed by Reddy et al.^54^ In brief, 5 × 10^4^ cells were plated in six-well plates and incubated for 24 h, followed by treatment with the respective components. After completion of the exposure time, the cells were trypsinized and centrifuged for 15 min at 1500 rpm. The obtained pellets were washed twice with phosphate-buffered saline (PBS) buffer, followed by centrifugation for 15 min at 1500 rpm. The pellet was resuspended in 100 μL of 1× Annexin binding buffer, and then cells were conjugated with 5 μL of Annexin V Alexa Fluor 488 and 1 μL of PI. Cells were mixed moderately and incubated at room temperature (RT) for 15 min in the dark. After 15 min of incubation, 400 μL of 1× Annexin-binding buffer was added, mixed gently, and kept on ice. The assay results were obtained by flow cytometry (BD FACSCelesta, New Jersey).

## 3 Results

### 3.1 Characterization of ZrO_2_/rGO NCs

#### 3.1.1 SEM and Energy-Dispersive X-ray Spectroscopy (EDS) Studies

**Figure 1:**
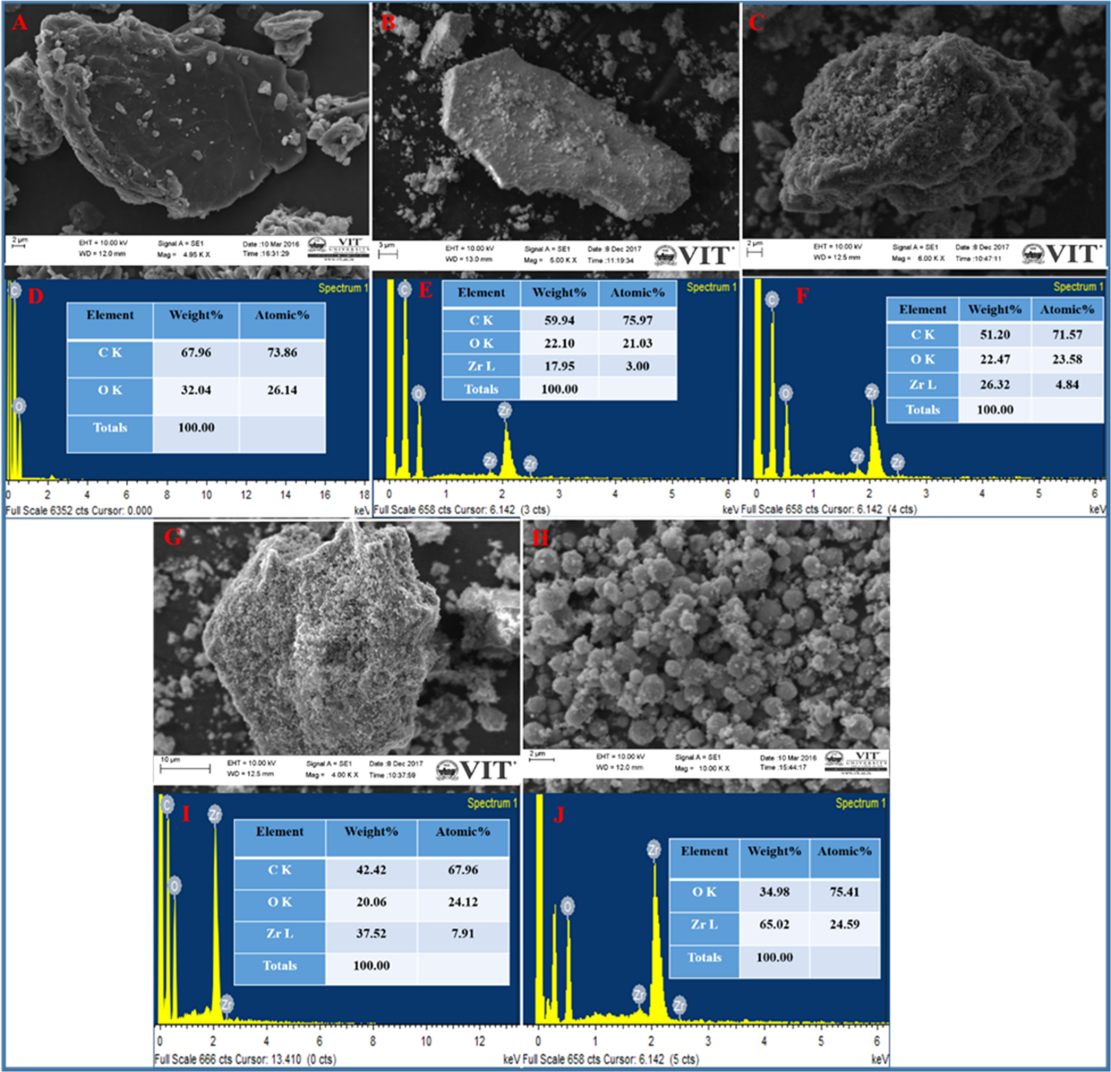
SEM images of rGO-AP (A), ZrO_2_/rGO NC 0.01M (B), ZrO_2_/rGO NC 0.05M (C), ZrO_2_/rGO NC 0.1M (G), and ZrO_2_ NP 0.05M (H). EDS patterns of rGO-AP (D), ZrO_2_/rGO NC 0.01M (E), ZrO_2_/rGO NC 0.05M (F), ZrO_2_/rGO NC 0.1M (I), and ZrO_2_ NP 0.05M (J).

The surface morphology and structural features of the as-prepared rGO-AP nanosheets and ZrO_2_/rGO NCs with varying Zr^4+^ ion concentrations were analyzed using a scanning electron microscope (SEM). rGO-AP showed the characteristic ruffled morphology consisting of a thin wrinkled structure and a sheet-like arrangement of rGO sheets with a thickness of 3–4 nm (Figure 1A). Figure 1B,C,G show the morphologies of 0.01, 0.05, and 0.1M ZrO_2_/rGO, respectively, revealing a large quantity of ZrO_2_ NP homogeneously decorated on the surface of rGO with the particle size ranging up to a few micrometers. The overall morphology of the ZrO_2_/rGO NCs reveals a uniform decoration of ZrO_2_ NPs, which covers the entire region of the rGO sheets. The SEM image of pure ZrO_2_ NP 0.05M (Figure 1H) exhibits a densely packed/assembled network of ZrO_2_ NPs. Furthermore, the EDS spectrum of the rGO sheets displays the corresponding carbon and oxygen signals (Figure 1D), whereas the EDS spectrum of pure ZrO_2_ NP (Figure 1J) exhibits the signals for zirconium and oxygen. The EDS spectrum of ZrO_2_/rGO NCs shows the signals for C, O, and Zr, suggesting the successful incorporation of ZrO_2_ NP onto the surface of rGO sheets (see Figure 1E,F,I). Note that according to the results, the obtained products do not possess any other components besides C, O, and Zr, indicating the purity of the synthesized nanomaterials. The respective weight and atomic percentages in of rGO-AP and ZrO_2_/rGO NC samples are presented in Table 1. The results suggest that with the increasing doping concentration of Zr^4+^ ions, the weight fraction of the ZrO_2_ NP concentration also increases within the respective samples, i.e., 17.95, 26.32, and 37.52% in ZrO_2_/rGO (0.01M), ZrO_2_/rGO (0.05M), and ZrO_2_/rGO (0.1M) NCs, respectively (see Table 1).

**Table 1:** Elemental (C, O, and Zr) Weight % and Atomic % of the Respective Nanocomposites (rGO-AP, ZrO_2_/rGO NC 0.01M, ZrO_2_/rGO NC 0.05M, ZrO_2_/rGO NC 0.1M, and ZrO_2_ NP 0.05M)

sample	C (wt %)	C (atom %)	O (wt %)	O (atom %)	Zr (wt %)	Zr (atom %)
rGO-AP	67.96	73.86	32.04	26.14		
ZrO_2_/rGO NC 0.01M	59.94	75.97	22.10	21.03	17.95	03.00
ZrO_2_/rGO NC 0.05M	51.20	71.57	22.47	23.58	26.32	04.84
ZrO_2_/rGO NC 0.1M	42.42	67.96	20.06	24.12	37.52	07.91
ZrO_2_ NP 0.05M			34.98	75.41	65.02	24.59

#### 3.1.2 TEM and SAED Studies

Also, a thin paper-like structure with very thin layers confirms the formation of rGO-AP nanosheets and the exfoliation of GO during the solvothermal process.

**Figure 2:**
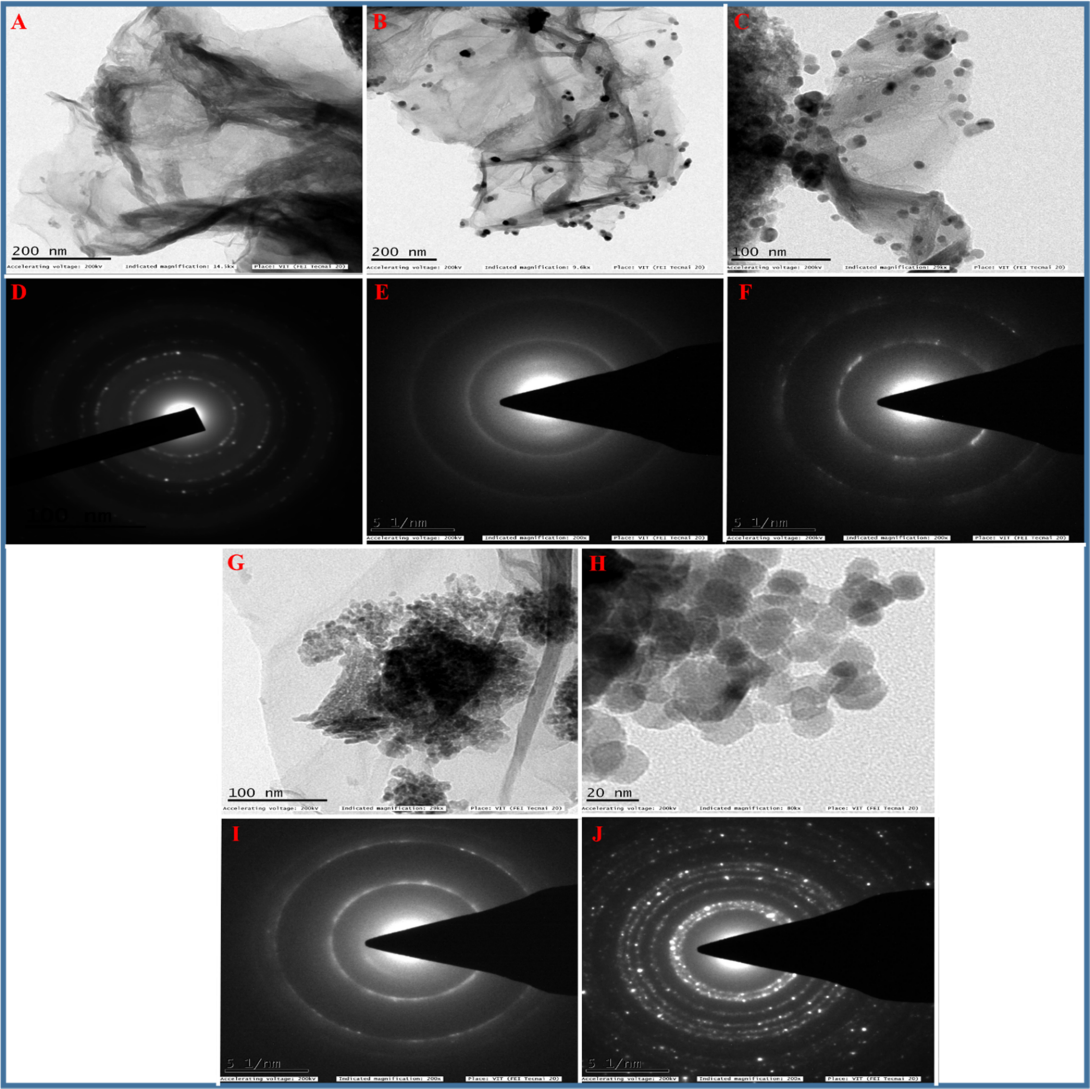
HR-TEM images of rGO-AP (A), ZrO_2_/rGO NC 0.01M (B), ZrO_2_/rGO NC 0.05M (C), ZrO_2_/rGO NC 0.1M (G), and ZrO_2_ NP 0.05M (H). SAED patterns of rGO-AP (D), ZrO_2_/rGO NC 0.01M (E), ZrO_2_/rGO NC 0.05M (F), ZrO_2_/rGO NC 0.1M (I), and ZrO_2_ NP 0.05M (J).

For TEM images of the ZrO_2_/rGO NCs (0.01, 0.05, and 0.1M), see Figure 2B,C,G, respectively. A more detailed structural analysis of the rGO-AP, ZrO_2_/rGO NCs, and ZrO_2_ NPs was performed using TEM (see Figure 2). Figure 2A shows a typical HR-TEM micrograph of the rGO-AP sample, revealing a rippled and crumpled morphology and the clear homogeneous dispersion of the ZrO_2_ NPs on the surface of rGO with sizes ranging from a few nanometers to a few tens of nanometers, indicating the uniform hybridization of rGO with ZrO_2_ NP.^56^ These results suggest that the applied solvothermal process provides an excellent microenvironment for rapid nucleation and growth of ZrO_2_ NPs, which is of prime importance for obtaining small NPs. The good dispersion of the ZrO_2_ NPs on the rGO surface could also act as a spacer, thus preventing the aggregation of ZrO_2_ NPs as well as restacking of the rGO sheets, which increases the stability of the fully exfoliated rGO sheets.^57^ The TEM image of pure ZrO_2_ NP exhibits aggregated NPs due to their smaller size and large surface energy when compared with ZrO_2_/rGO NCs (Figure 2H). The corresponding selected area electron diffraction (SAED) patterns of rGO-AP, ZrO_2_/rGO NCs, and ZrO_2_ NPs are shown in Figure 2D–F,I,J. Figure 2D depicts the SAED pattern of rGO-AP, and the discontinuous dotted circles observed on the crystalline carbon indicate the characteristic feature of fewer graphene layers with a typical hexagonal symmetry. Figure 2E,F,I,J illustrates the corresponding SAED patterns for ZrO_2_/rGO NCs (0.01, 0.05, and 0.1M) and ZrO_2_ NPs (0.05M), respectively. The SAED pattern clearly shows spherical aggregated ZrO_2_ NPs, which represent the high nanocrystallinity of the formed product. The concentric Debye–Scherrer rings can be indexed to the (101), (110), (200), (211), and (220) reflecting planes and match well with those of the tetragonal ZrO_2_ structure, which is in good agreement with the XRD data of the tetragonal phases of ZrO_2_ nanocrystals (see Supporting Information).

#### 3.1.3 X-ray Photoelectron Spectroscopic Studies

**Figure 3:**
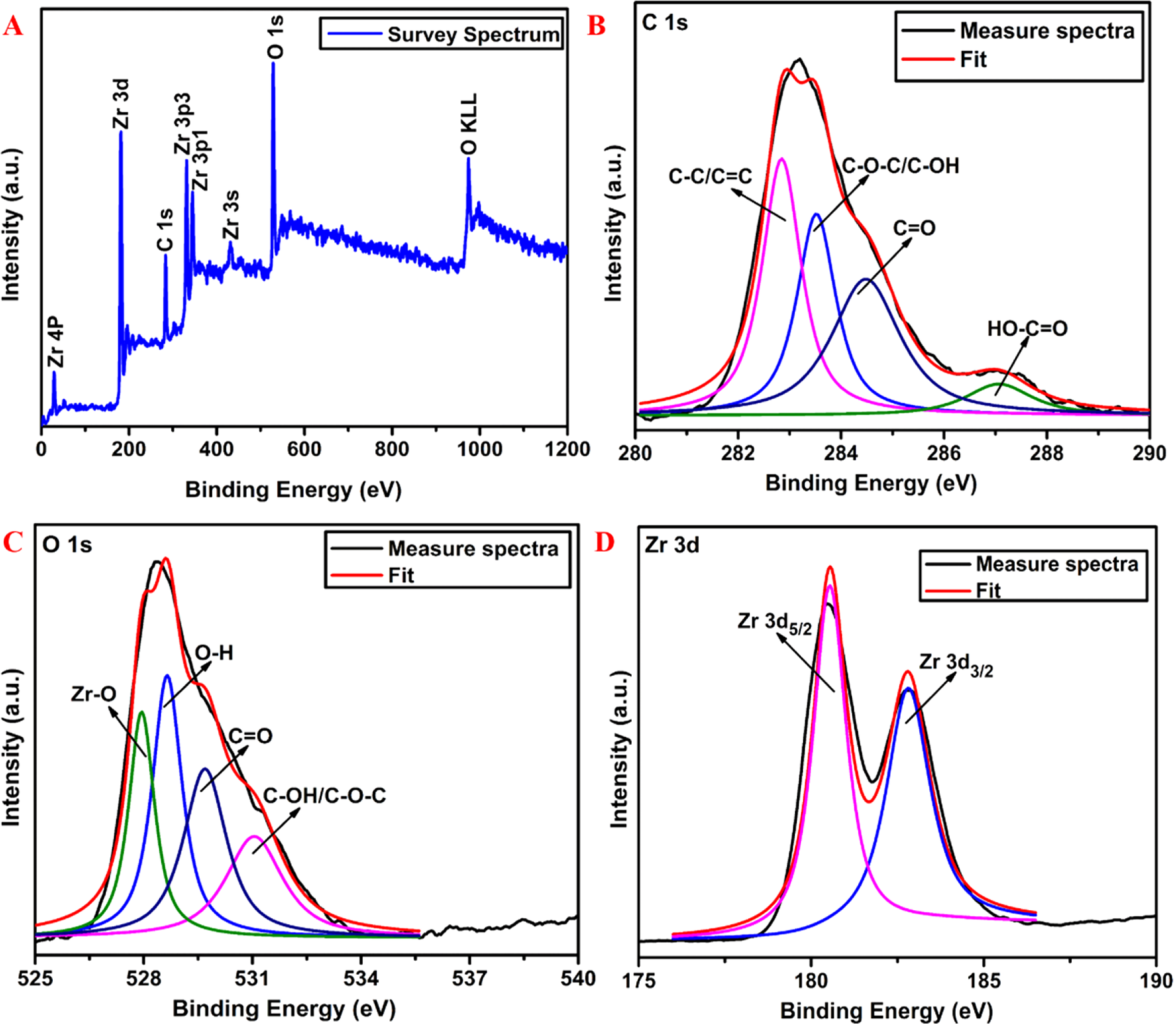
XPS survey spectra (A), C 1s region (B), O 1s region (C), and high-resolution spectra of the Zr 3d region (D) of ZrO_2_/rGO NC 0.05M.

To investigate the elemental composition of the ZrO_2_/rGO NC (0.05M) sample, XPS measurements were performed in the specified range from 0 to 1200 eV (Figure 3).

Figure 3A shows the XPS survey spectra of the sample and confirms the existence of elements belonging to ZrO_2_ and carbon, i.e., C 1s, O 1s, and Zr 3d. This result is in agreement with the EDS elemental analysis (see Figure 1E,F,I above). The signal for the core levels of C 1s obtained for the ZrO_2_/rGO NC (0.05M) sample (see Figure 3B) exhibits four types of functionalized carbon atoms: C–C/C=C (282.8 eV), C–O–C/C–OH (hydroxyl and epoxy groups, 283.5 eV), C=O (carbonyl groups, 284.4 eV), and HO–C=O (287.0 eV). The formed rGO exhibits a weaker intensity of oxygen-containing functional groups compared with GO, and therefore, an increase in the C–C peak intensity indicates the successful reduction of GO to rGO.^58^ The O 1s core levels of the sample (see Figure 3C) show three main components related to C–OH/C–O–C (531.0 eV), C=O (529.7 eV), and O–H (528.6 eV). In addition to the three carbon–oxygen bonding peaks, the spectra also show an additional peak at 527.9 eV belonging to Zr–O (Figure 3C). The shift in the C 1s and O 1s peaks of ZrO_2_/rGO NCs is due to Zr donating some electrons to O and C. The expected Zr 3d peaks (see Figure 3D) located at 180.5 (Zr-C) and 182.8 eV (Zr-OH) can be attributed to the spin–orbit splitting of the Zr 3d components, Zr 3d_5/2_ and Zr 3d_3/2_, respectively, which indicates the fully oxidized zirconium ions in their Zr^4+^ state.^59,60^ All of these results confirm that the one-pot solvothermal synthesis method favors the formation of ZrO_2_ NPs followed by the simultaneous reduction of GO within the ZrO_2_/rGO NCs.

### 3.2 Effect of ZrO_2_/rGO NCs on the Viability of Cancer Cells

**Figure 4:**
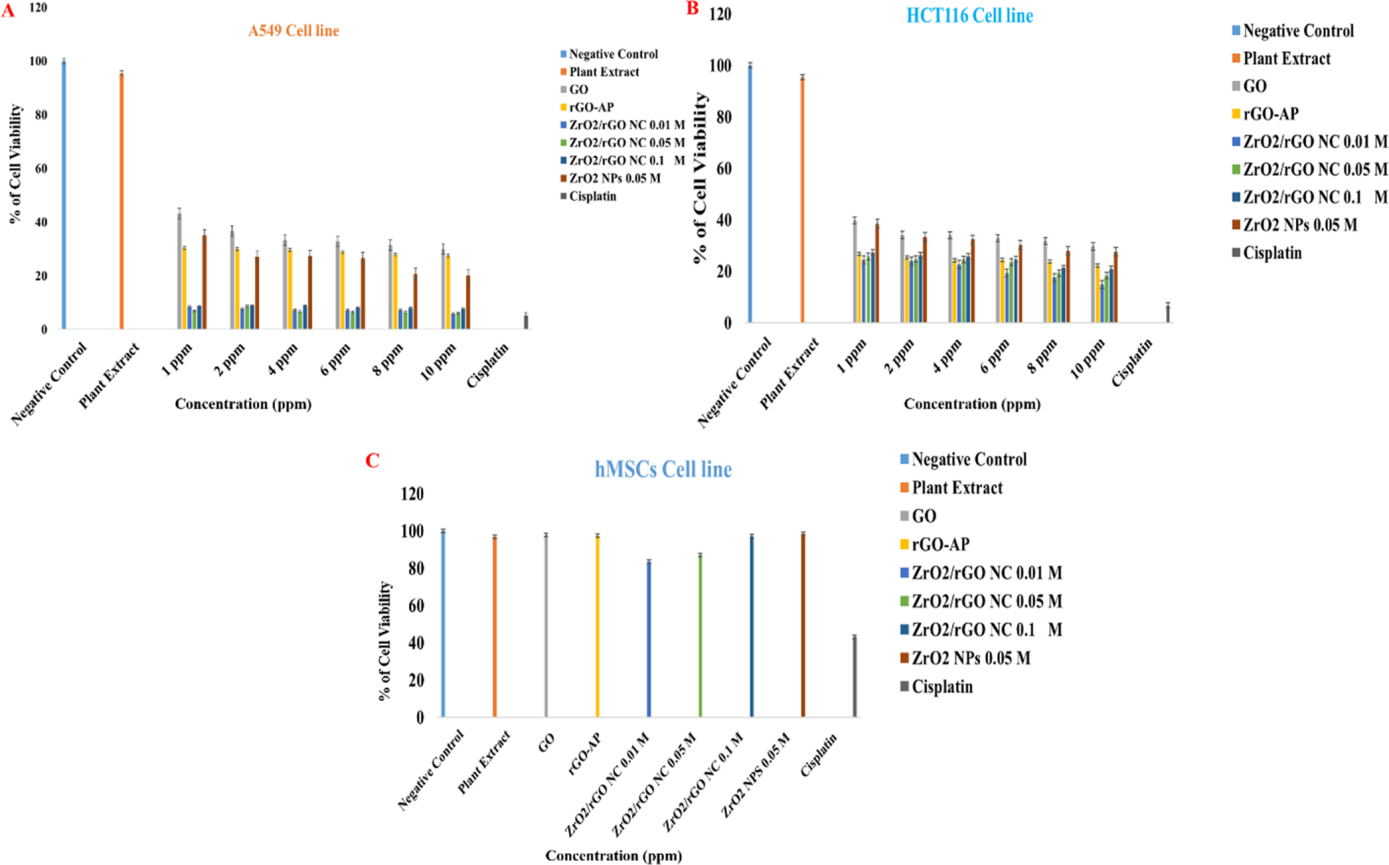
Cytotoxicity effects of GO, rGO-AP, ZrO_2_/rGO NC 0.01M, ZrO_2_/rGO NC 0.05M, ZrO_2_/rGO NC 0.1M, and ZrO_2_ NP 0.05M on the A549 cancer cell lines (A), HCT116 cell line (B), and normal cell line of hMSCs (C). All of the studied nanomaterials used the optimal concentration of 10 ppm. Data are expressed as mean ± standard error (SE) of three independent runs for each item (*p < 0.05).

The cell viabilities of human cancer cell lines A549 and HCT116 and one normal cell line of hMSCs were evaluated by the MTT assay and flow cytometry protocols after 24 h exposure of the cell lines to different concentrations of ZrO_2_-rGO NCs, as described in the Experimental Section. The MTT assay results demonstrate that both the cancer cell lines, A549 and HCT116, exhibit an increase in cytotoxic activity with increasing dosage of nanocomposites, whereas these nanocomposites did not show any adverse effects on the normal cell line of hMSCs (see Figure 4).

#### 3.2.1 Cell Viability of A549 Cells after Exposure to ZrO_2_/rGO NCs

The highest cell viability of 95.43% is recorded when cell lines are exposed to the plant extract, whereas GO and rGO-AP exhibit a significant reduction in cell viability (%) in a dose-dependent manner for 1, 2, 4, 6, 8, and 10 ppm (43.16–29.9% for GO and 30.43–27.43% for rGO-AP on the A549 cancer cell line, respectively, *p < 0.05; see Figure 4A). After the exposure of the A549 cancer cell line to different doses of ZrO_2_/rGO NC 0.01M, the cell viabilities (%) are 8.53, 7.66, 7.16, 7.23, 7.23, and 5.83%, whereas they are 6.86, 8.6, 6.7, 6.46, 6.33, and 6.2% for ZrO_2_/rGO NC 0.05M and 8.63, 8.93, 8.96, 8.16, 8.0, and 7.83% for ZrO_2_/rGO NC 0.1M exposure, respectively.

These results suggest that the cell viability (%) decreases with an increasing ZrO_2_/rGO NC dose (see Figure 4). At the maximum dose concentration of 10 ppm, the positive control drug cisplatin shows the highest cytotoxic effect (5.26% cell viability). On treatment with 1, 2, 4, 6, 8, and 10 ppm pure ZrO_2_ NPs, 35.0, 27.0, 27.27, 26.53, 20.6, and 20.1% viabilities of A549 cancer cells were observed, respectively (see Figure 4A) (Table 2).

**Table 2:** MTT Assay of the Anticancer Activity of the Respective Nanocomposites (GO, rGO-AP, ZrO_2_/rGO NC 0.01M, ZrO_2_/rGO NC 0.05M, ZrO_2_/rGO NC 0.1M, ZrO_2_ NP 0.05M along with the Negative Control, Plant Extract, and Positive Control Drug Cisplatin toward A549 Cell Lines)

A549 cell lines									
	negative control	plant extract	1 ppm	2 ppm	4 ppm	6 ppm	8 ppm	10 ppm	cisplatin
negative control	100								
plant extract		95.43							
GO			43.16	36.75	33.26	32.7	31.36	29.9	
rGO-AP			30.43	29.98	29.7	28.76	27.9	27.43	
rGO/ZrO_2_ 0.01M			8.53	7.66	7.16	7.23	7.23	5.83	
rGO/ZrO_2_ 0.05M			6.86	8.6	6.7	6.46	6.33	6.2	
rGO/ZrO_2_ 0.1M			8.63	8.93	8.96	8.16	8	7.83	
ZrO_2_ NPs 0.05M			35	27	27.26	26.53	20.6	20.1	
cisplatin									5.26

#### 3.2.2 Cell Viability of HCT116 Cells after Exposure to ZrO_2_/rGO NCs

The cell viability (%) of human colon cancer cells HCT116 was monitored after exposure to GO, rGO-AP, and ZrO_2_/rGO NCs. The cell viabilities (%) are 39.73, 34.07, 33.97, 32.83, 31.73, and 29.66% for GO whereas 26.73, 25.43, 24.17, 24.47, 23.8, and 22.21% for rGO-AP at exposure dosage concentrations of 1, 2, 4, 6, 8, and 10 ppm, respectively. Similarly, cell viabilities (%) of HCT116 colon cancer cells are 38.47, 33.37, 32.33, 30.27, 28.0, and 27.63% after exposure to ZrO_2_ NP (1, 2, 4, 6, 8, and 10 ppm) (see Figure 4B). According to Figure 4, human HCT116 colon cancer cells exhibit higher cytotoxic activity than the human A549 cancer cell line after exposure to ZrO_2_/rGO NCs. The obtained MTT results primarily confirm that the cell viabilities of human HCT116 colon cancer cell lines are 24.4, 23.93, 22.67, 19.43, 17.53, and 14.93% after exposure to ZrO_2_/rGO NC 0.01M of 1, 2, 4, 6, 8, 10 ppm concentrations, respectively, which is significantly low (*p < 0.05). The cell viabilities (%) of human HCT116 colon cancer cell lines are 25.77, 24.73, 24.57, 23.57, 19.27, and 18.33%, respectively, after exposure to ZrO_2_/rGO NC 0.05M for the same set of dosage concentrations. However, the cell viabilities (%) of HCT116 cell lines after exposure to the same set of concentrations of ZrO_2_/rGO NC 0.1M are 27.23, 26.13, 25.73, 24.7, 21.23, and 20.8%, which is slightly higher than that of ZrO_2_/rGO NC 0.05M. Interestingly, the cell viability (%) is found to be slightly higher when exposed to ZrO_2_/rGO NCs prepared with high doping concentrations of ZrO_2_ NP on the rGO surface (see Figure 4) (Table 3) because the observed hydrodynamic sizes were slightly higher for higher doping concentrations.

**Table 3:** MTT Assay for Anticancer Activity of the Respective Nanocomposites (GO, rGO-AP, ZrO_2_/rGO NC 0.01M, ZrO_2_/rGO NC 0.05M, ZrO_2_/rGO NC 0.1M, ZrO_2_ NP 0.05M along with the Negative Control, Plant Extract, and Positive Control Drug Cisplatin toward HCT116 Cell Lines)

HCT116 cell lines									
	negative control	plant extract	1 ppm	2 ppm	4 ppm	6 ppm	8 ppm	10 ppm	cisplatin
negative control	100								
plant extract		95.43							
GO			39.73	34.06	33.96	32.83	31.73	29.66	
rGO-AP			26.73	25.43	24.16	24.46	23.8	22.21	
rGO/ZrO_2_ 0.01M			24.4	23.93	22.66	19.43	17.53	14.93	
rGO/ZrO_2_ 0.05M			25.76	24.73	24.56	23.56	19.26	18.33	
rGO/ZrO_2_ 0.1M			27.23	26.13	25.73	24.7	21.23	20.8	
ZrO_2_ NPs 0.05M			38.46	33.36	32.33	30.26	28	27.63	
cisplatin									6.73

### 3.3 Cell Viability of Normal hMSCs after Exposure to ZrO_2_/rGO NCs

Along with cancer cell lines, the cytotoxic effect of the as-synthesized nanomaterials at the optimum concentration (10 ppm) on normal human cell lines (hMSCs) was also investigated. The obtained results show that these nanocomposites do not have any significant toxicity toward normal cell lines (hMSCs) (cell viabilities of 97.71, 97.41, 83.40, 87.06, 97.26, and 98.47% after exposure to GO, rGO-AP, ZrO_2_/rGO NC 0.01M, ZrO_2_/rGO NC 0.05M, ZrO_2_/rGO NC 0.1M, and ZrO_2_ NP 0.05M, respectively) in contrast to the positive control drug cisplatin (43.07%) (Figure 4C, Table 4).

**Table 4:** MTT Assay for the Anticancer Activity of the Respective Nanocomposites (GO, rGO-AP, ZrO_2_/rGO NC 0.01M, ZrO_2_/rGO NC 0.05M, ZrO_2_/rGO NC 0.1M, ZrO_2_ NP 0.05M along with the Negative Control, Plant Extract, and Positive Control Drug Cisplatin toward hMSCs Cell Lines (10 ppm))

hMSCs cell lines (10 ppm)									
	negative control	plant extract	GO	rGO-AP	rGO/ZrO_2_ 0.01M	rGO/ZrO_2_ 0.05M	rGO/ZrO_2_ 0.1M	ZrO_2_ NPs 0.05M	cisplatin
negative control	100								
plant extract		96.80							
GO			97.71						
rGO-AP				97.41					
rGO/ZrO_2_ 0.01M					83.40				
rGO/ZrO_2_ 0.05M						87.06			
rGO/ZrO_2_ 0.1M							97.26		
ZrO_2_ NPs 0.05M								98.47	
cisplatin									43.07

These results clearly demonstrate that the ZrO_2_/rGO NCs obtained by one-pot solvothermal synthesis are toxic to cancer cell lines but biocompatible with normal cell lines. Moreover, the IC_50_ values of GO, rGO-AP, ZrO_2_/rGO NCs (0.01, 0.05, and 0,1M), and ZrO_2_ NP are presented in Table S2. The IC_50_ values obtained for HCT116 and A549 cell lines after exposure to ZrO_2_-rGO NC (0.01M) are 0.7921 and 1.3898 μg L_,_^–1^ respectively, which are lower than those in other reports.

### 3.4 Flow Cytometry Study for the Measurement of Cell Viability

**Figure 5:**
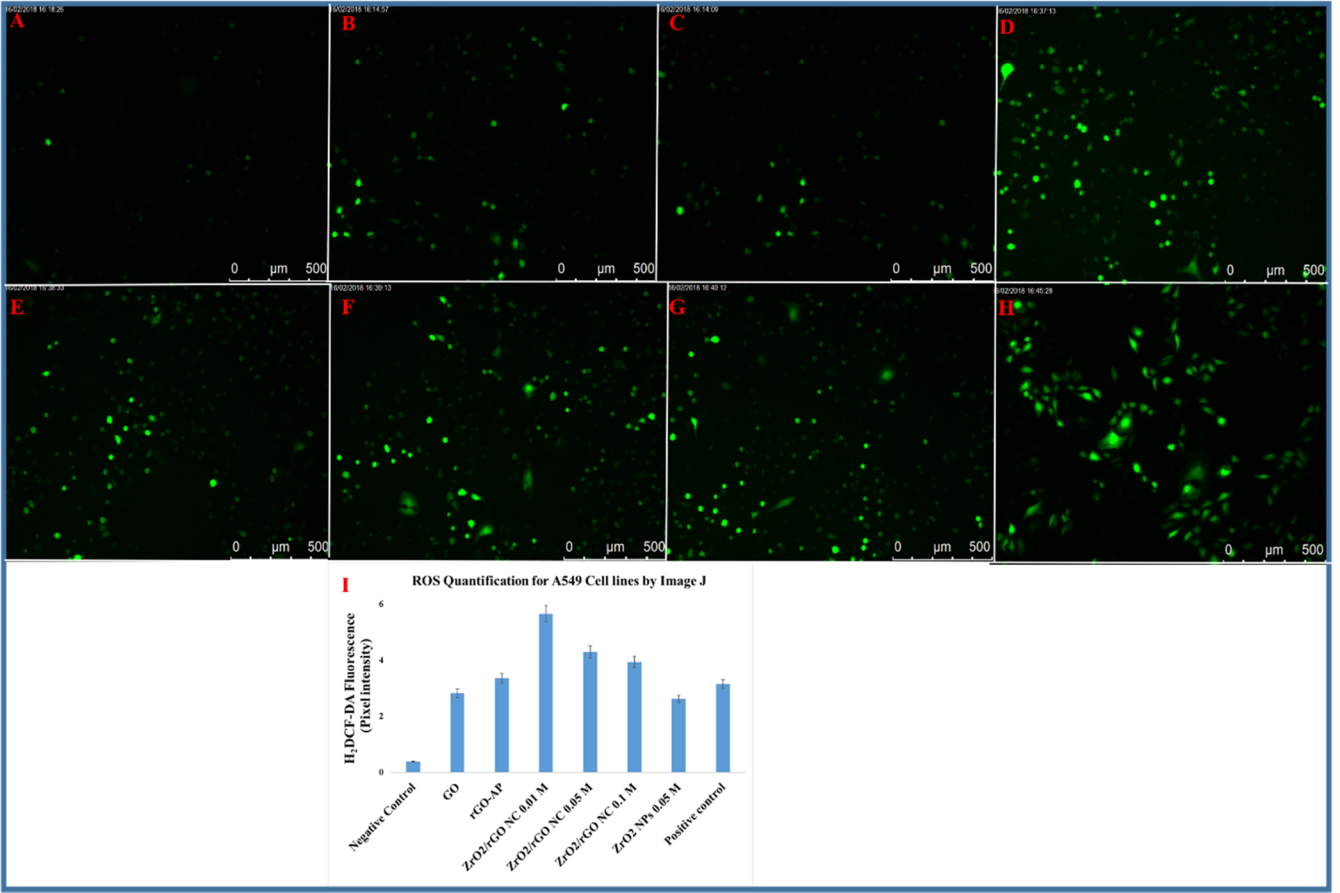
Representative fluorescence microscopic images of ROS generation for the A549 cancer cell line: negative control (A), GO (B), rGO-AP (C), ZrO_2_/rGO NC 0.01M (D), ZrO_2_/rGO NC 0.05M (E), ZrO_2_/rGO NC 0.1M (F), ZrO_2_ NP 0.05M (G), and positive control drug (cisplatin) (H). Images are representative of three independent experiments. Quantification of the mean fluorescence intensity was performed using Image J from three images from each run from different groups (I). Data are the average ± SE of three independent runs conducted in triplicate wells in each run (*p < 0.05).

**Figure 6:**
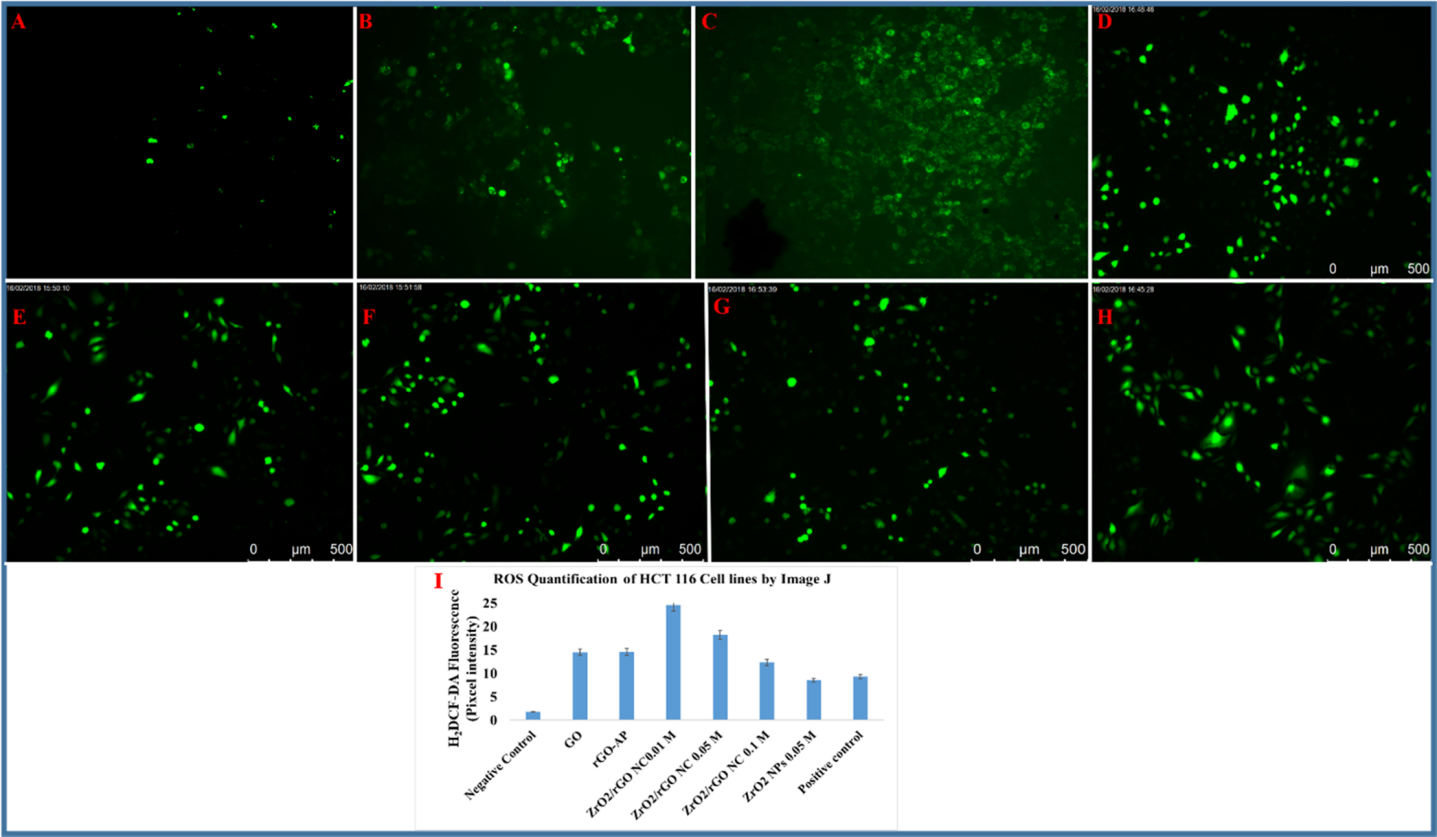
Representative fluorescence microscopic images of ROS generation for the HCT116 cancer cell line: negative control (A), GO (B), rGO-AP (C), ZrO_2_/rGO NC 0.01M (D), ZrO_2_/rGO NC 0.05M (E), ZrO_2_/rGO NC 0.1M (F), ZrO_2_ NP 0.05M (G), and positive control drug (cisplatin) (H). Images are representative of three independent experiments. (I) Quantification of the mean fluorescence intensity was performed using Image J from three images from each run from different groups. Data are average ± SE of three independent runs conducted in triplicate wells in each run (I) (*p < 0.05).

**Figure 7:**
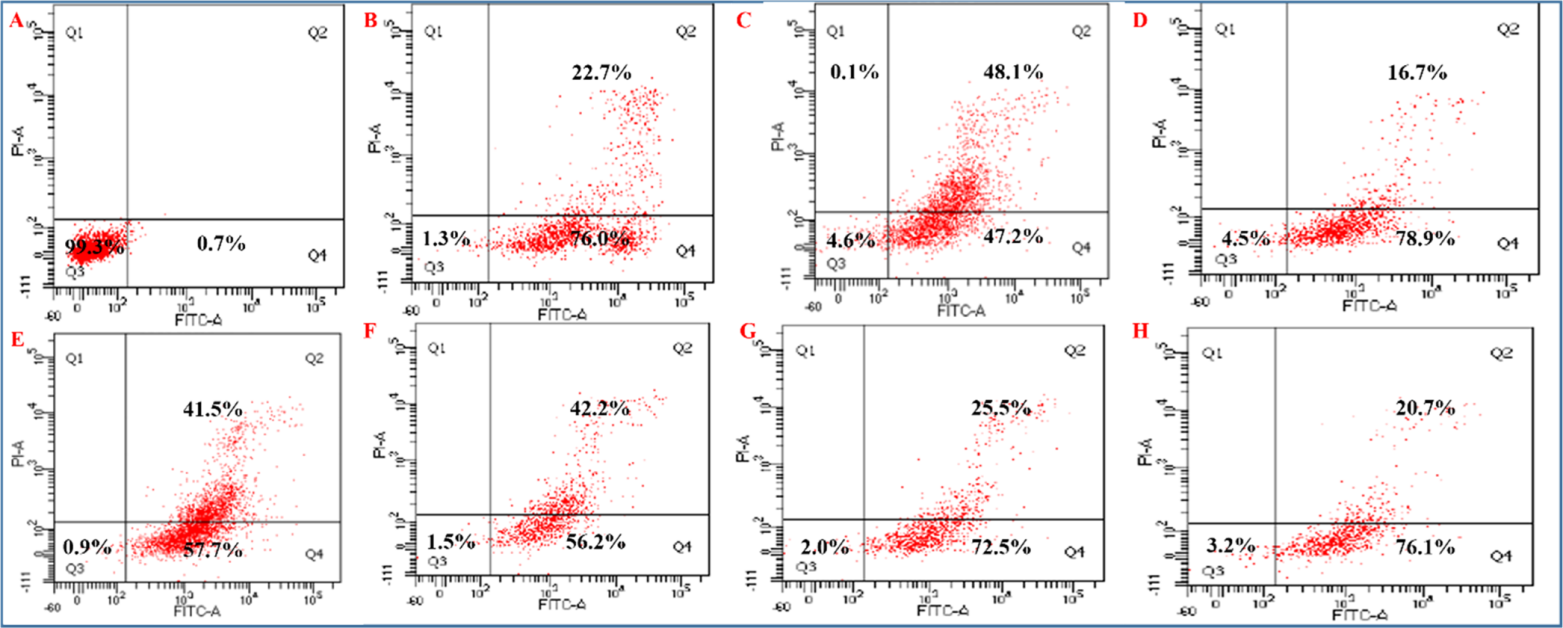
Flow cytometric analysis of apoptotic cell death after treatment with the maximum concentration of (10 ppm) ZrO_2_/rGO NCs exposed to A549 cell lines for 24 h and double labeling with Annexin V-FITC and PI. Scatter diagrams of cells exposed to the respective compounds are shown in (A–H) as follows: negative control (A), positive control drug (cisplatin) (B), GO (C), rGO-AP (D), ZrO_2_/rGO NC 0.01M (E), ZrO_2_/rGO NC 0.05M (F), ZrO_2_/rGO NC 0.1M (G), and ZrO_2_ NP 0.05M (H). In the flow cytogram, cells are denoted as follows: Q3, live cells; Q4: apoptotic cells; Q2, late apoptotic cells, and Q1, necrotic cells. Data are representative of three independent experiments.

The flow cytometry results illustrate a cell viability of 99.3% for human A549 lung cancer cells with reference to the negative control. For the other components such as GO, rGO-AP, ZrO_2_/rGO NC 0.01M, ZrO_2_/rGO NC 0.05M, ZrO_2_/rGO NC 0.1M, and ZrO_2_ NP 0.05M, the cell viability decreased to 4.6, 4.5, 0.9, 1.5, 2.0, and 3.2%, respectively. The positive control drug cisplatin showed a cell viability of 1.3% (see Figures 5–7 and Table S3).

**Figure 8:**
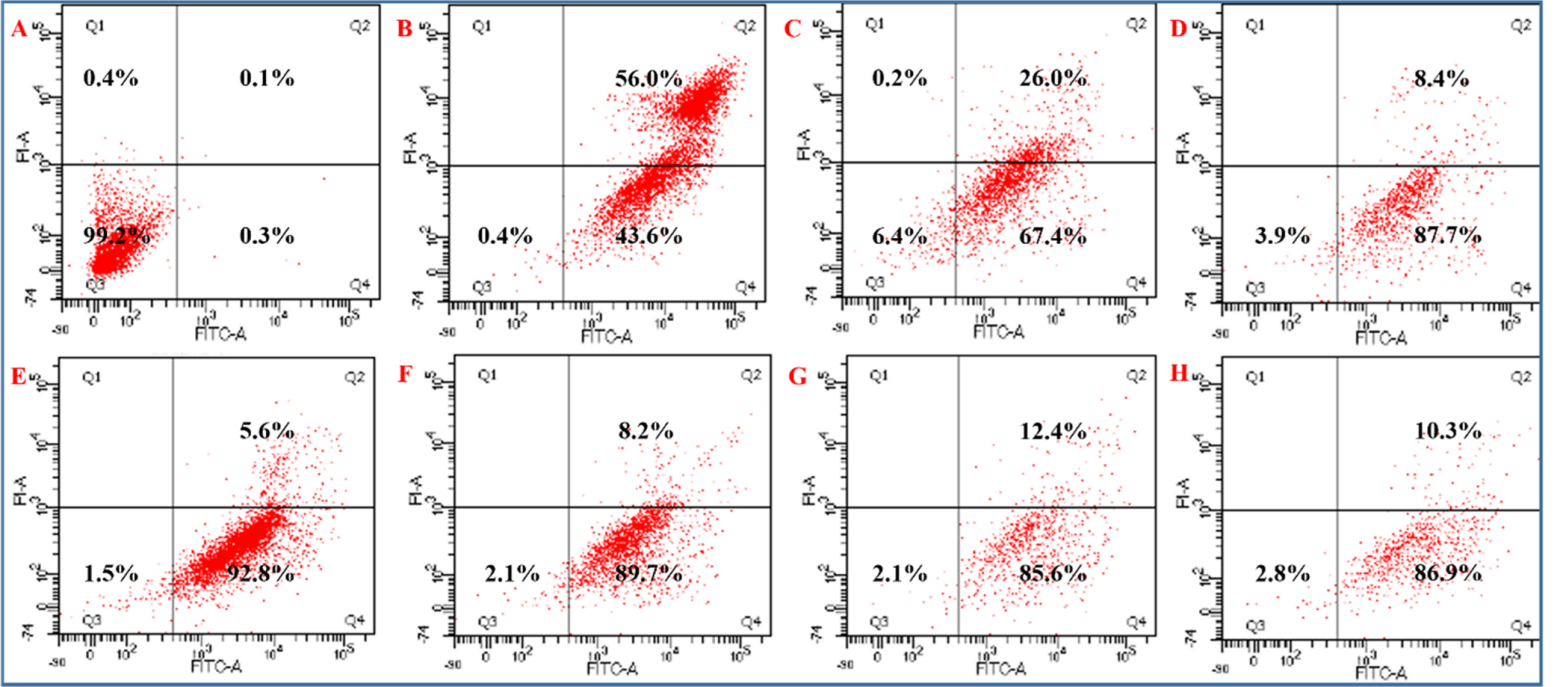
Flow cytometric analysis of apoptotic cell death after treatment with the maximum concentration of (10 ppm) ZrO_2_/rGO NCs exposed to HCT116 cell lines for 24 h and double labeling with Annexin V-FITC and PI. Scatter diagrams of cells exposed to the respective compounds are shown in (A–H) as follows: negative control (A), positive control (cisplatin) (B), GO (C), rGO-AP (D), ZrO_2_/rGO NC 0.01M (E), ZrO_2_/rGO NC 0.05M (F), ZrO_2_/rGO NC 0.1M (G), and ZrO_2_ NP 0.05M (H). In the flow cytograms, cells are denoted as follows: Q3, region denotes live cells; Q4, apoptotic cells; Q2, late apoptotic cells; and Q1, necrotic cells. Data are representative of three independent experiments.

The flow cytometry results further demonstrate that the negative control showed 99.2% viability to human HCT116 colon cancer cells. Other groups, i.e., GO, rGO-AP, ZrO_2_/rGO NC 0.01M, ZrO_2_/rGO NC 0.05M, ZrO_2_/rGO NC 0.1M, and ZrO_2_ NP 0.05M, show cell viabilities (%) of 6.4, 3.9, 1.5, 2.1, 2.1, and 2.8%, respectively, after 24 h of exposure, whereas the positive control drug cisplatin showed 0.4% cell viability (Figure 8 and Table S5). Based on the above cytotoxicity results, we chose the highest concentration (10 ppm) for further experiments (see Figure 4).

### 3.5 Role of ZrO_2_/rGO NCs in Reactive Oxygen Species (ROS) Generation

Several reports have highlighted that different nanomaterials tend to initiate oxidative stress in the cellular environment by generating reactive oxygen species (ROS) that induce cytotoxicity.^61,62^ The oxidative stress potential of the as-synthesized materials GO, rGO-AP, ZrO_2_/rGO NCs (0.01, 0.05, and 0.1M), and ZrO_2_ NP 0.05M were tested against A549, HCT116, and normal cell lines (hMSCs). The results correlate with the cell viability studies above, i.e., higher ROS levels and cellular oxidative stress are found in the order of ZrO_2_/rGO NC 0.01M > ZrO_2_/rGO NC 0.05M > ZrO_2_/rGO NC 0.1M > ZrO_2_ NP 0.05M > rGO-AP > GO for cancer cell lines (A549 and HCT116). Especially, ROS generation is observed to be more in HCT116 cell lines compared with A549 cell lines (see Figures 5 and 6). Image J software is utilized to determine the significant increase in ROS by ∼3.0, ∼3.5, ∼3.0, ∼4.0, ∼4.5, and ∼6.0-fold for GO, rGO-AP, ZrO_2_ NP 0.05M, ZrO_2_/rGO NC 0.1M, ZrO_2_/rGO NC 0.05M, and ZrO_2_/rGO NC 0.01M, respectively. When compared with the standard drug cisplatin, the synthesized ZrO_2_/rGO NC 0.01M exhibits ∼6.0-fold increased ROS generation. In the case of HCT116 cells, the ROS generation is increased by ∼24.5, ∼18.2, ∼12.3, ∼8.5, ∼14.5, and ∼14.5-fold for ZrO_2_/rGO NC 0.01M, ZrO_2_/rGO NC 0.05M, ZrO_2_/rGO NC 0.1M, ZrO_2_ NP 0.05M, rGO-AP, and GO, respectively (see Figure 6). Compared with cisplatin, ZrO_2_/rGO NC 0.01M shows increased ROS generation by ∼24.5-fold, where the mean fluorescent intensity is compared with the control (see Table S3, Figures 5I and 6I). The nanocomposites studied here do not show any ROS generation in normal cell lines (hMSCs), which indicates that no cytotoxic effect is observed in normal cells (see Figure S8).

## 4 Discussion

The advancement of anticancer therapeutic agents has tuned their ability to induce both cytotoxicity and apoptosis in cancer cell lines. These materials comprise the hotspot in cancer treatment. In this study, we evaluated the anticancer therapeutic activity of one-pot solvothermally synthesized ZrO_2_ NPs decorated on the surface of reduced graphene oxide (ZrO_2_/rGO NCs) toward human cancer cell lines A549 (lung cancer), HCT116 (colorectal cancer), and one normal cell line hMSCs (umbilical cord blood-derived).

Initially, the physicochemical characterization of the synthesized ZrO_2_/rGO NCs was performed using different instrumental techniques such as FTIR, XRD, SEM, EDS, TEM, SAED, XPS spectroscopy, and dynamic light scattering (DLS) to determine parameters such as functional groups, formed crystal structure, shape, size, purity, hydrodynamic size, agglomeration, and aqueous stability. The FTIR spectroscopy technique reveals that oxygen-containing moieties such as carboxyl, hydroxyl, and epoxy groups were reduced from the GO surface and converted to rGO-AP via a biological reduction process (Figure S1B). XRD analysis shows that the GO peak intensity decreased after the successful reduction of GO to rGO-AP using aqueous *A. paniculata* leaf extract as a green reducing and stabilizing agent, which indicates that the oxygen-containing functional groups present on the GO surface are effectively removed/reduced. In the case of ZrO_2_/rGO NCs, the tetragonal phase of ZrO_2_ nanocrystals was decorated on the rGO surface (Figure S1A).

SEM and TEM results illustrate that the formed ZrO_2_ NPs are mostly spherical in shape and are homogeneously distributed throughout the surface of rGO. EDS data indicate that the synthesized GO, rGO-AP, and ZrO_2_/rGO NCs consist of C, O, and Zr as major elements without any other unexpected elements, indicating the high purity of the synthesized nanocomposites. The SAED pattern of the spherical aggregated ZrO_2_ NPs indicates that they are highly crystalline, and the concentric Debye–Scherrer rings, i.e., diffraction rings that are indexed to the (101), (110), (200), (211), and (220) crystal planes, confirm the tetragonal structure of ZrO_2_ NPs. Whenever the synthesized GO, rGO-AP, and ZrO_2_/rGO NCs were introduced into biological buffer mediums such as PBS, cell culture medium, and water, the sizes of these nanocomposites increased approximately 5–10 times compared with their initial size. These changes occur mainly due to the interactions between the ZrO_2_/rGO NCs and the biological components present in the cell growth media, which have the ability to impact the agglomeration/precipitation of these nanocomposites. As a result, it ultimately leads to lower ζ-potential values recorded in PBS and cell culture medium when compared with water medium (see Table S1 and Figures S2–S7).

Cytotoxicity studies play a key role in identifying the anticancer activity level of any compound. According to the present study, low cytotoxicity levels are shown by raw components such as GO, rGO-AP, and ZrO_2_ NPs when compared with different doped ZrO_2_/rGO NCs (0.01, 0.05, and 0.1M) and the positive control drug cisplatin. Furthermore, other NCs such as ZrO_2_/rGO NCs 0.01M/0.05M showed anticancer activity equivalent to that of the cisplatin drug at higher dosage concentrations (6, 8, and 10 ppm: 6.46, 6.33, 6.2 vs 5.26%, respectively). Similar results are obtained from the Annexin V assay for ZrO_2_/rGO NC 0.01M (A549 vs HCT116: 0.9 vs 1.5%), ZrO_2_/rGO NC 0.05M (A549 vs HCT116: 1.5 vs 2.1%), and ZrO_2_/rGO NC 0.1M (A549 vs HCT116: 2.0 vs 2.1), which suggests a higher anticancer effect on the HCT116 cell line than on the A549 cell line. These results demonstrate that ZrO_2_/rGO NCs have significant (*p < 0.05) cytotoxicity toward both A549 and HCT116 cancer cell lines (Figures 7 and 8). Also, we chose only three different specific doping concentrations of ZrO_2_/rGO (0.01M (Low), 0.05M (Medium), and 0.1M (High)), where ZrO_2_NPs are uniformly deposited onto the rough solid surface of reduced graphene oxide (rGO). The major purpose of this study is to perform an in-depth analysis of the behavior of the synthesized nanocomposite materials toward two different cancer cell lines, including A549 and HCT116, and a noncancer cell line of hMSCs. From the results, it can be clearly observed that lower concentrations of ZrO_2_NP-doped nanocomposites are very effective in exhibiting higher anticancer activity when compared with other doped concentrations (Medium and High) because the ZrO_2_NP-doped composites of lower concentration are smaller in size, almost spherical in shape, and well separated from each other so that they can easily penetrate into the cellular organelles, whereas medium and high concentrations of ZrO_2_NP-doped composites are bulky in nature and are clustered and so these NPs cross the barriers at a slower rate and exhibit lower anticancer activity.

To determine the statistical significance between two different cell lines, we executed a two-tailed paired Student’s *t*-test assuming the absolute *t*-value to be 2.23 at a degree of freedom (df) of 10 and a probability (*p*) of 0.05 on experimental treatment data. We calculated the *t*-value to determine the validity of the null hypothesis for the two treated groups among A549 and HCT116 cells. The *t*-values of rGO-AP ZrO_2_/rGO NCs (0.01, 0.05, and 0.1M) and pure ZrO_2_ NP 0.05M-treated A549 cell lines are 2.71, 6.75, 13.84, 13.31, and 2.85, respectively, which are higher than the absolute *t*-value of 2.23 (see Figure 4 and Table S6A). This in turn suggests that all of the treated groups of A549 cell lines are statistically significant with respect to the GO-treated group. Similarly, the calculated *t*-values for all of the groups of HCT116 cells and the recorded *t*-values for rGO-AP, ZrO_2_/rGO NCs (0.01, 0.05, and 0.1M), and pure ZrO_2_ NP 0.05M are 6.06, 6.33, 5.83, 5.31 and 0.92, respectively. Note that all of the observed values are found to be higher than the absolute *t*-value of 2.23, except the value of pure ZrO_2_ NP 0.05M (see Figure 4 and Table S6B). Hence, the ZrO_2_ NP 0.05M-treated group is not significantly different from the GO-treated group, but all other groups are statistically different. Then, we performed a Student’s *t*-test to determine the probability (*p*) value, and all p-values are much lower than 0.05, which indicates that all of the treatment groups are statistically significant (Figure 4 and Table S6B). However, all treated groups associated with hMSCs are not statistically different (Table S6C) as *t*-values of all treated groups are lower than the absolute *t*-value. This result suggests further that hMSCs are not affected by the exposure of the studied nanomaterials.

Reactive oxygen species (ROS) are the natural derivative agents of cellular oxidative metabolism and play various roles in cellular activities such as cell death, cell survival, differentiation cell signaling, and inflammation. When cells are exposed to NPs, they interact with cell membranes and assist in the transfer of ionic signals to different cell organelles followed by the accelerated generation of excessive ROS.^63^ Finally, higher ROS generation leads to more oxidative stress, which results in more cell death.

Based on several reports on nanotoxicology,^64^ both graphene and semiconductor nanomaterials such as ZrO_2_ NPs are highly effective in elevating the levels of ROS within the cellular environment, which ultimately results in cell death. Hence, in the present study, we utilized both the combinations of graphene and ZrO_2_ NP decorated on rGO NCs to test their anticancer activity. In the present study, the generation of ROS drastically increased with ZrO_2_/rGO NC treatment. Especially, it was ∼14.5-fold higher than that of cisplatin in HCT116 cells (see Figure 6). Other nanocomposites also significantly increased the ROS levels (see Figures 5 and 6). The generation of different levels of ROS shows variable oxidative stress, which correlates with the concentration of nanomaterials exposed to the cells.^68^ This correlation is noticed in the present study at the high optimum dosage concentration (10 ppm), i.e., a high level of generated ROS causes more cancer cell death. This study proves that the synthesized NCs show good cytotoxic effect toward cancer cells and no toxicity toward normal cells. However, in-depth anticancer cellular mechanisms need to be investigated.

The flow-cytometry results further demonstrate that after the treatment of human A549 lung cancer cells with different concentrations of nanocomposites, ROS were produced in the cell environment, which caused apoptotic cell death (see Figure 7). HCT116 cancer cells exposed to GO, rGO-AP, ZrO_2_/rGO NC 0.01M, ZrO_2_/rGO NC 0.05M, ZrO_2_/rGO NC 0.1M, and ZrO_2_ NP 0.05M show 67.4, 87.7, 92.8, 89.7, 85.6, and 86.9% apoptotic cell death, respectively. These results suggest that ZrO_2_ NP-doped rGO NCs produce enough ROS, which increase the apoptotic cell death (see Figure 8). Similarly, A549 cancer cells exposed to GO, rGO-AP, ZrO_2_/rGO NC 0.01M, ZrO_2_/rGO NC 0.05M, ZrO_2_/rGO NC 0.1M, and ZrO_2_ NP 0.05M show 47.2, 78.9, 57.7, 56.2, 72.5, and 76.1% apoptotic cell death, respectively (see Figure 7). It is interesting that A549 cancer cells are less prone to apoptotic cell death than HCT116 cancer cells, but A549 cells are highly prone to late apoptotic cell death (20.7–41.5%) compared with HCT116 (5.6–10.3%) (see Figures 7 and 8). Further studies are needed to confirm these observations and the actual mechanism of apoptotic cell death by measuring caspase-3 and caspase-9 activation for early-stage and mid-stage apoptotic cell death. However, cytochrome C for DNA fragmentation and nuclear collapse would be studied using a TUNEL assay to analyze late apoptotic cell death in an upcoming project, which will be helpful to elucidate the actual reasons for the different responses of A549 and HCT116 cancer cells to the above-treated NCs during early-stage and late-stage apoptotic cell death.

In addition, Figure S8 shows the fluorescence microscopic images of ROS generation after exposure of the different studied nanocomposites to normal cell lines (hMSCs), which suggest that no ROS is generated after this treatment. The results of this study clearly indicate that ZrO_2_ NP-doped rGO NCs and ZrO_2_ NPs are nontoxic to normal cells (hMSCs), confirming their potential for applications in biomedical and cancer therapy.

## 5 Conclusions

In conclusion, we demonstrated that ZrO_2_/rGO NCs can be successfully synthesized via a one-pot solvothermal green synthetic method and characterized well by different physical and chemical techniques. Based on the current results, these ZrO_2_/rGO NCs exhibit distinct anticancer effects toward human HCT116 and A549 cancer cell lines, while they do not induce any adverse effect on normal cells (hMSCs). In addition, the observed cytotoxic effect is more pronounced and mediated via oxidative stress by the generated ROS in a manner similar to that of chemotherapeutic drugs, which induce/trigger apoptosis. ZrO_2_/rGO NCs exhibit ∼14.5-fold efficiency, which was higher than that of cisplatin in HCT116 cells, and also, the apoptotic rate was found to be 86.9% in the case of HCT116 cells. Hence, considering these potentially exciting properties, ZrO_2_/rGO NCs could serve as potential anticancer agents in cancer therapy.

## Supporting Information Available

Spectral characterization of different samples; hydrodynamic size distributions and ζ-potentials of GO, rGO-AP, ZrO_2_/rGO NC 0.01M, ZrO_2_/rGO NC 0.05M, ZrO_2_/rGO NC 0.1M, and ZrO_2_ NP 0.05M; representative fluorescence microscopic images of ROS generation; ζ-potential and DLS analysis; IC_50_ values (μg L^–1^) of nanomaterials; ROS-quantified mean fluorescence intensity (MFI) data values; data obtained from FITC-conjugated Annexin-V and PI staining assay for A549 cells and HCT116 cells; statistical analysis of data sets for significant evaluation of the anticancer activity of ZrO_2_/rGO NCs on A549, HCT116, and hMSC cell lines by an MTT assay (PDF)
