# Supplementary material for: Efficient One-Pot Solvothermal Synthesis and Characterization of Zirconia Nanoparticle-Decorated Reduced Graphene Oxide Nanocomposites: Evaluation of Their Enhanced Anticancer Activity toward Human Cancer Cell Lines
Source: ACS Omega. 2023 Jan 3;8(2):2406–20. doi: 10.1021/acsomega.2c06822 (PMC9850483; doi:10.1021/acsomega.2c06822)
Supplement: Supplementary file 1 — ao2c06822_si_001.pdf [file ao2c06822_si_001.pdf]

Supplementary Information

An efficient one-pot Solvothermal synthesis and characterization of zirconia nanoparticles decorated reduced graphene oxide nanocomposites: Evaluation of its enhanced anticancer activity towards human cancerous cell lines

Nalinee Kanth Kadiyala [a], Badal Kumar Mandal [a,*], L. Vinod Kumar Reddy [b], Crispin H.W. Barnes[c], Luis De Los Santos Valladares[c,d,e,*], Dwaipayan Sen [b]

[a]*Trace Elements Speciation Research Laboratory, Department of Chemistry, School of Advanced Sciences, Vellore Institute of Technology (VIT), Vellore 632014, India*

[b]*Cellular and Molecular Therapeutics Laboratory, Centre for Biomaterials, Cellular and Molecular Theranostics, Vellore Institute of Technology (VIT), Vellore 632014, India*

[c] *Cavendish Laboratory, Department of Physics, University of Cambridge, Cambridge CB3 0HE, United Kingdom*

[d] *Faculty of Physics and Technology, L.N. Gumilyov Euroasian National University, Nur-Sultan, Kazakhstan 010000*

[e] *Laboratorio de Cerámicos y Nanomateriales, Facultad de Ciencias Físicas, Universidad Nacional Mayor de San Marcos, Ap. Postal 14-0149 Lima, Peru*

*Corresponding Authors.

Dr. Badal Kumar Mandal, Tel.:+914162202339; Fax: +914162243092; E-mail address: badalmandal@vit.ac.in, badalkmandal@gmail.com

L De Los Santos Valladares, email ld301@cam.ac.uk

Brief statement

- Spectral characterization of different samples
- Hydrodynamic size distribution and Zeta potential of GO
- Hydrodynamic size distribution and Zeta potential of rGO-AP
- Hydrodynamic size distribution and Zeta potential of ZrO_2/rGO NC 0.01M
- Hydrodynamic size distribution and Zeta potential of ZrO_2/rGO NC 0.05M
- Hydrodynamic size distribution and Zeta potential of ZrO_2/rGO NC 0.1M
- Hydrodynamic size distribution and Zeta potential of ZrO_2 NP 0.05M
- Representative fluorescence microscopic images of ROS generation
- Zeta potential and DLS analysis; IC_{50} Values (µg L^{-1}) of nanomaterials
- ROS quantified mean fluorescence intensity (MFI) data values

- Data obtained from FITC-conjugated Annexin-V and PI staining assay for A549 cells
- Data obtained from FITC-conjugated Annexin-V and PI staining assay for HCT116 cells
- Statistical analysis of data sets for significant evaluation of anticancer activity of ZrO_2/rGO NCs on A549 cell lines by MTT assay
- Statistical analysis of data sets for significant evaluation of anticancer activity of ZrO_2/rGO NCs on HCT116 cell lines by MTT assay
- Statistical analysis of data sets for significant evaluation of anticancer activity of ZrO_2/rGO NCs on hMSCs cell lines by MTT assay

Results

XRD Studies:

X-ray diffraction studies were employed to identify the crystal structure and determining the diffraction pattern of all the synthesized nanocomposites such as GO, rGO-AP, ZrO_2/rGO NC of 0.1 0.05, 0.01and ZrO_2 NP are represented in Figure S1A, where GO formation was confirmed with diffraction patterns at 10.25° and 42.30°, which were attributed to the d_{002} plane of GO and 100 carbon planes of a hexagonal structure, respectively (Fig. S1A (a)).[1] The identified d-spacing values of GO were 8.62 A$^{\circ}$ and 2.13 A$^{\circ}$ respectively. After complete reduction of GO to rGO-AP, there was a significant reduction in the diffraction intensity was observed and moreover, two new diffraction peaks were identified at 25.63° and 42.96°, which correspond to the rGO-AP d_{002} plane with a d-spacing values of 3.41 A$^{\circ}$ and 2.09 A$^{\circ}$ respectively. This pattern confirms the successful reduction of GO to rGO and interestingly, the d-spacing was reduced from GO to rGO, the reason might be the removal/loss of oxygen functionalities during the reduction of GO (Fig. S1A (b)). Also, the tetragonal-ZrO_2 NPs formation was further confirmed with the observed diffraction pattern at 30.2 (101), 35.17 (110), 50.6 (200), 60.3 (211), and 74.6 (220) A^{0} and this pattern was well-matched with the JCPDS card no: 88-1007. All these diffractions of ZrO_2 were observed in nanocomposites, however there were no rGO peaks found in the composite pattern, this might be due to the

uniform distribution of ZrO_2 in between the graphene layers and absence of rGO influence on the crystalline phase growth pattern of ZrO_2 during the calcination of composite.[2]

FT-IR spectroscopy studies:

FT-IR analysis result reveals the functional groups existed on the surface of the nanomaterials. Fig. S1B represents the FT-IR spectra of GO, rGO-AP, and ZrO_2/rGO NCs (Fig. S1B (a-f)). Broad peak at 3200-3300 cm^{-1} region confirms the presence of O-H functional group on the surface of all the synthesized materials. The frequency region from 435-469 cm^{-1} illustrates the bonding between the Zirconium and oxygen moieties (Zr-O). GO shows the peaks at 1726, 1622, 1375, 1220, 1093 and 1053 cm^{-1} (Fig. S1B(a)), which are attributed to the stretching vibration of -C=O group, bending vibration of -O-H, and skeletal vibration of C-C group, bending vibration of O-H group of -C-OH molecule, C-O stretching frequency of ethers and epoxy molecules and stretching vibration of C-O-C group, respectively. The bands from 1670-1550 cm^{-1} may be due to the surface adsorbed hydroxyl or water molecules from the carbon materials.[3-5]

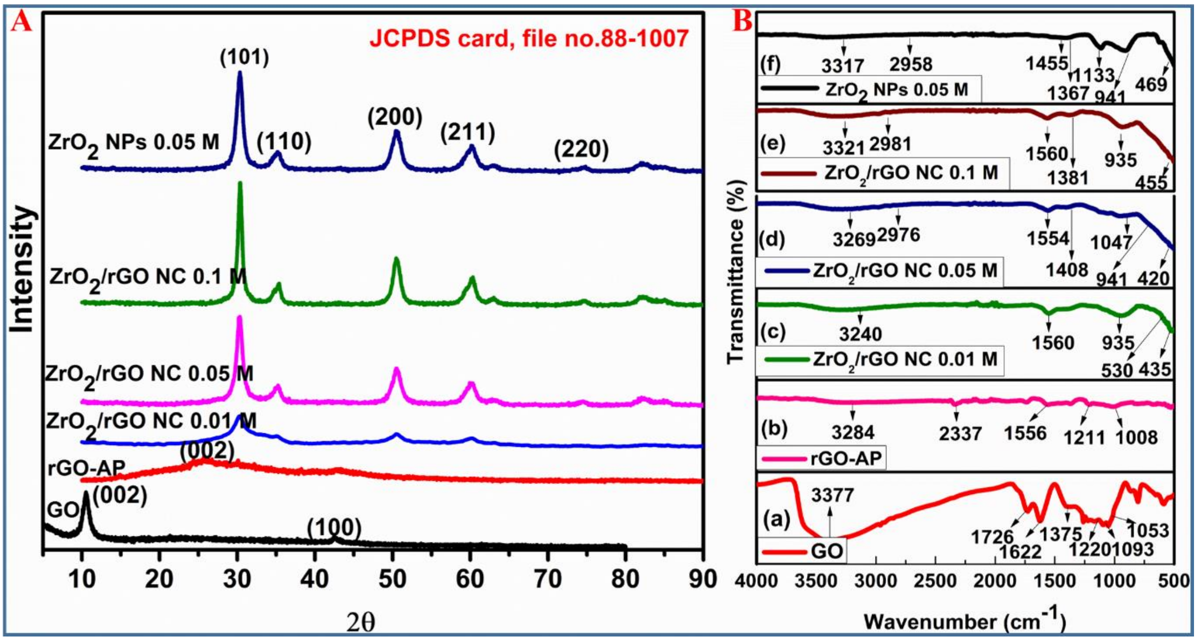

Figure S1: Spectral characterization of different samples. XRD patterns of GO (a), rGO-AP (b), ZrO_2/rGO NC 0.01M (c), ZrO_2/rGO NC 0.05M (d), ZrO_2/rGO NC 0.1M (e), and ZrO_2 NP

0.05M (f) (A); FTIR spectra of GO (a), rGO-AP (b), ZrO_2/rGO NC 0.01M (c), ZrO_2/rGO NC 0.05M (d), ZrO_2/rGO NC 0.1M (e), and ZrO_2 NP 0.05M (f)

DLS and Zeta potential studies:

It is well known that nanocomposites often have the ability to form the agglomerates in solution mediums, which shows further effect on their interactions with biological systems. Thus, the interaction behaviour of the above synthesized samples GO, rGO-AP, ZrO_2/rGO NC 0.01M, ZrO_2/rGO NC 0.05M, ZrO_2/rGO NC 0.1M, and ZrO_2 NP 0.05M when exposed to three different liquid environments like PBS, culture medium and water systems were tested using DLS (dynamic light scattering) analysis. By using this analysis, we determined the hydrodynamic diameter, zeta potential and polydispersity indices (PI) values of different suspensions as illustrated in Table S1 and Figure S2-S7. These synthesized ZrO_2/rGO NCs show excellent colloidal stability in aqueous system, but their stability would vary significantly whenever transferred into aqueous system containing biological buffers like Phosphate Buffer Solution (PBS) and DMEM (Cell culture medium). When we noticed from the above obtained results the exfoliation of all prepared ZrO_2/rGO NCs in water medium was very much stable and among all the NCs the highest value is recorded for rGO-AP having a zeta potential of -59.5 mV. Minor aggregations are observed for all the functionalized ZrO_2/rGO NCs' dispersions into PBS and cell culture medium. This is further accompanied by an increase in hydrodynamic diameters in cell culture medium when compared to those in water and PBS medium. The hydrodynamic size of ZrO_2 NP 0.05M is maximum as 4410.3 nm followed by GO as 4187.1 nm (Table S1). Consistently, the zeta potential is decreased in PBS and cell culture medium (Table S1). This phenomenon is mainly due to the cell growth medium was composed of many biological components like high protein content of serum, albumin, fibrinogen, amino acids, vitamins, and also the presence of phosphate have each previously been shown an impact on agglomeration/precipitation of NPs. These findings serve better to

identify the cause for the decrease in colloidal stability upon particle incubations in biological mediums such as in PBS and DMEM medium. Polydispersity indices (PI) is an autocorrelation function, which corresponds to the ratio of second moment and square of mean value of logarithm and it denotes the extent of size distribution.[6] The ZrO_2/rGO NC suspensions shows larger PI values with larger ionic strengths i.e., in DMEM (culture medium) and PBS when compared to water medium exhibiting lower ionic strengths (Table S1)

Table S1: Zeta potential and DLS analysis of GO, rGO-AP, ZrO_2/rGO NC 0.01M, ZrO_2/rGO NC 0.05M, ZrO_2/rGO NC 0.1M and ZrO_2 NP 0.05M in different solvent systems such as Phosphate Buffer solution (PBS), Cell culture medium and Water.

Sample	Solvent medium	Zeta (mV)	DLS (nm)	PDI
GO	PBS	-42.3	1601.3	0.301
GO	Cell culture medium	-27.9	4187.1	0.381
GO	Water	-45.8	1323.3	0.264
rGO-AP	PBS	-2.7	4.9	0.833
rGO-AP	Cell culture medium	-14.4	1982.3	0.185
rGO-AP	Water	-59.5	1446.2	0.141
ZrO_2/rGO NC 0.01M	PBS	-6.4	0.4	1.063
ZrO_2/rGO NC 0.01M	Cell culture medium	-3.9	0.4	1.224
ZrO_2/rGO NC 0.01M	Water	-37.8	2.0	1.639
ZrO_2/rGO NC 0.05M	PBS	-6.6	54.2	2.402
ZrO_2/rGO NC 0.05M	Cell culture medium	-5.9	2727.5	0.256
ZrO_2/rGO NC 0.05M	Water	-28.9	5.0	0.597
ZrO_2/rGO NC 0.1M	PBS	-2.3	39.2	2.757
ZrO_2/rGO NC 0.1M	Cell culture medium	-13.8	1409.6	0.141
ZrO_2/rGO NC 0.1M	Water	-52.6	3.2	1.581
ZrO_2 NP 0.05M	PBS	1.2	5109.1	0.469
ZrO_2 NP 0.05M	Cell culture medium	-4.4	4410.3	0.413
ZrO_2 NP 0.05M	Water	-31.9	4229.0	2.097

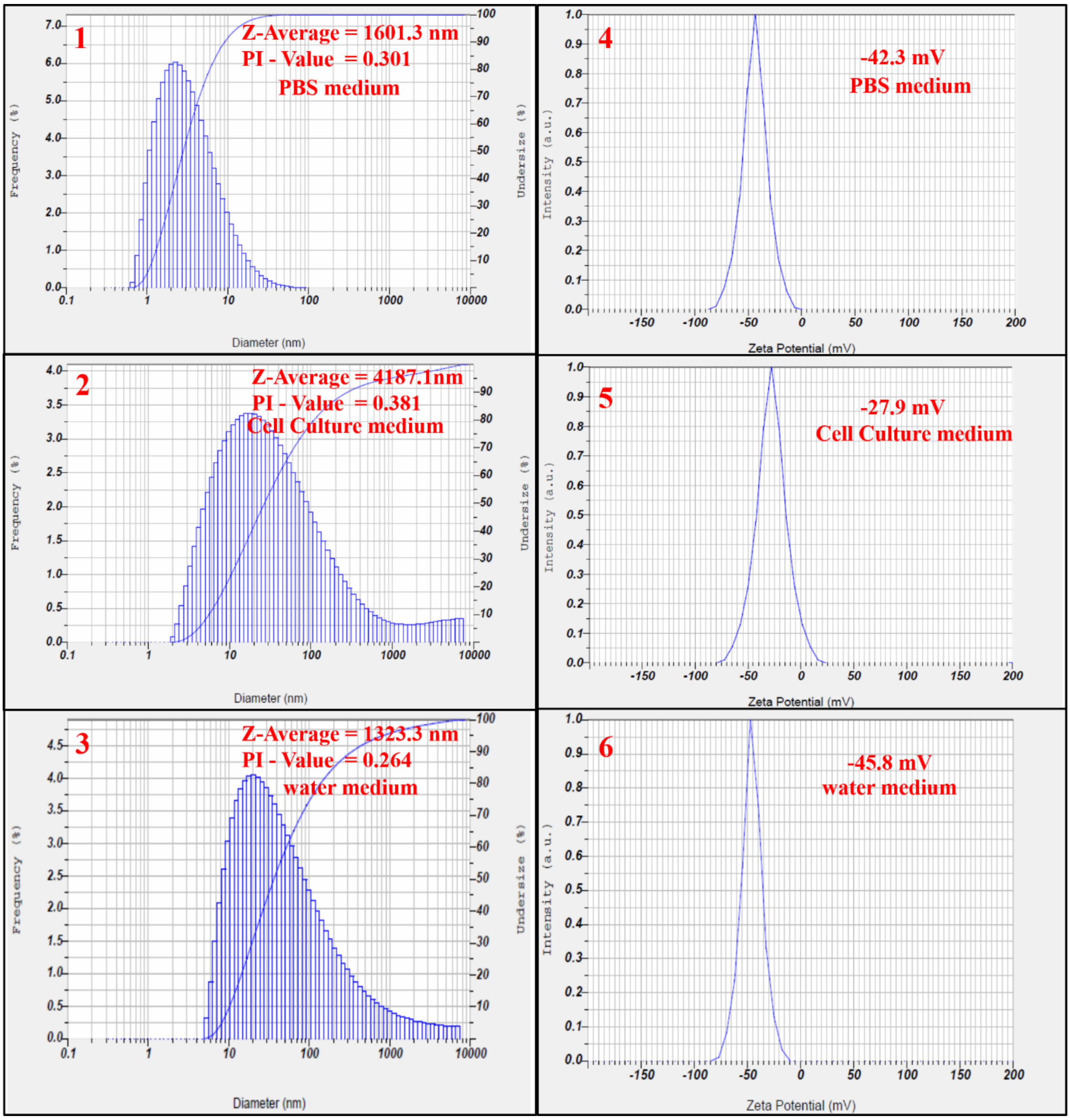

Figure S2. Hydrodynamic size distribution and Zeta potential of GO (0.5 mg mL^{-1}) in PBS medium (1, 4), Cell culture medium (2, 5) and Water medium (3, 6) were measured by DLS at room temperature.

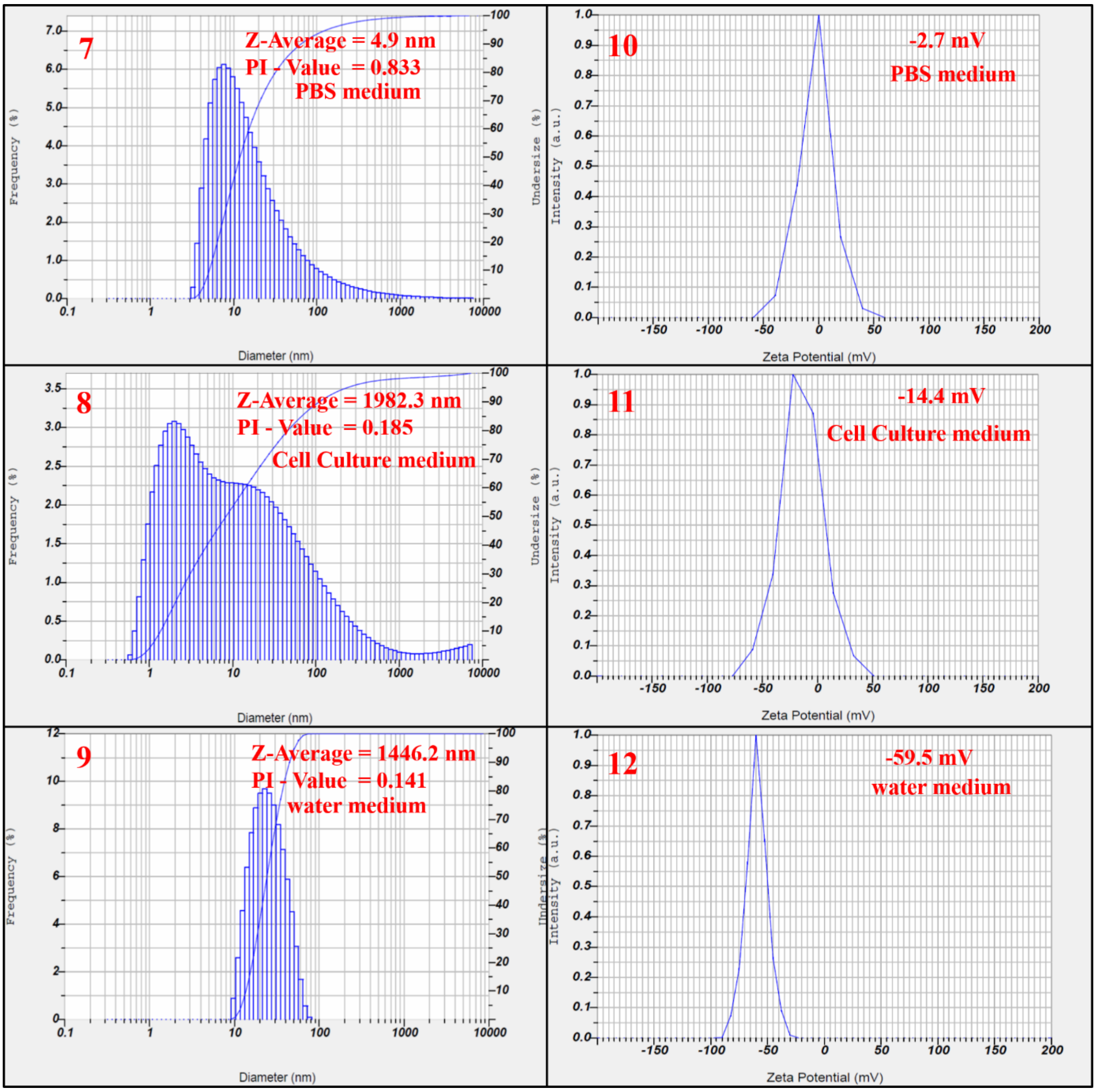

Figure S3. Hydrodynamic size distribution and Zeta potential of rGO-AP (0.5 mg mL^{-1}) in PBS medium (7, 10), Cell culture medium (8, 11) and Water medium (9, 12) were measured by DLS at room temperature.

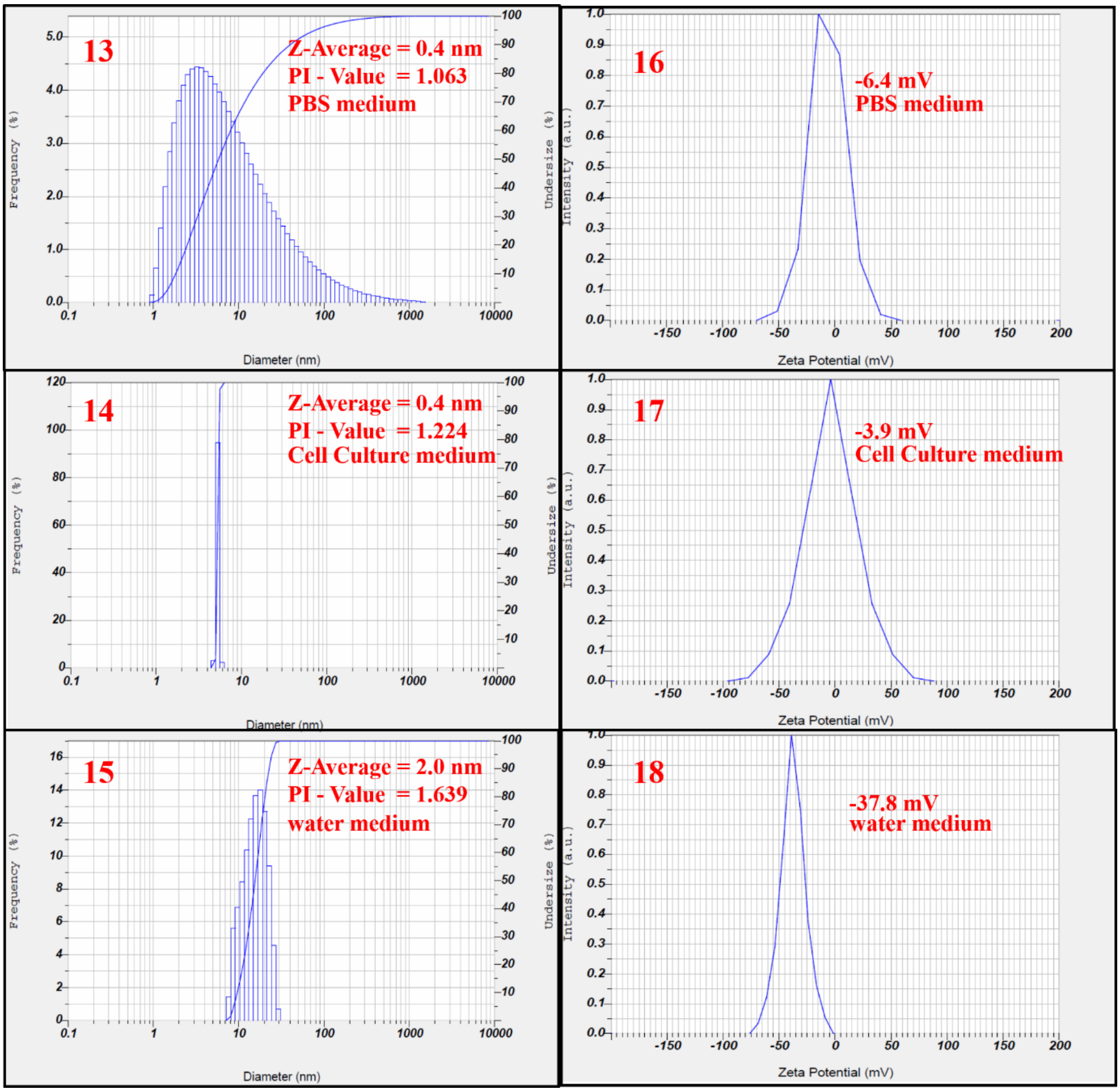

Figure S4. Hydrodynamic size distribution and Zeta potential of ZrO_2/rGO NC 0.01M (0.5 mg mL^{-1}) in PBS medium (13, 16), Cell culture medium (14, 17) and Water medium (15, 18) were measured by DLS at room temperature.

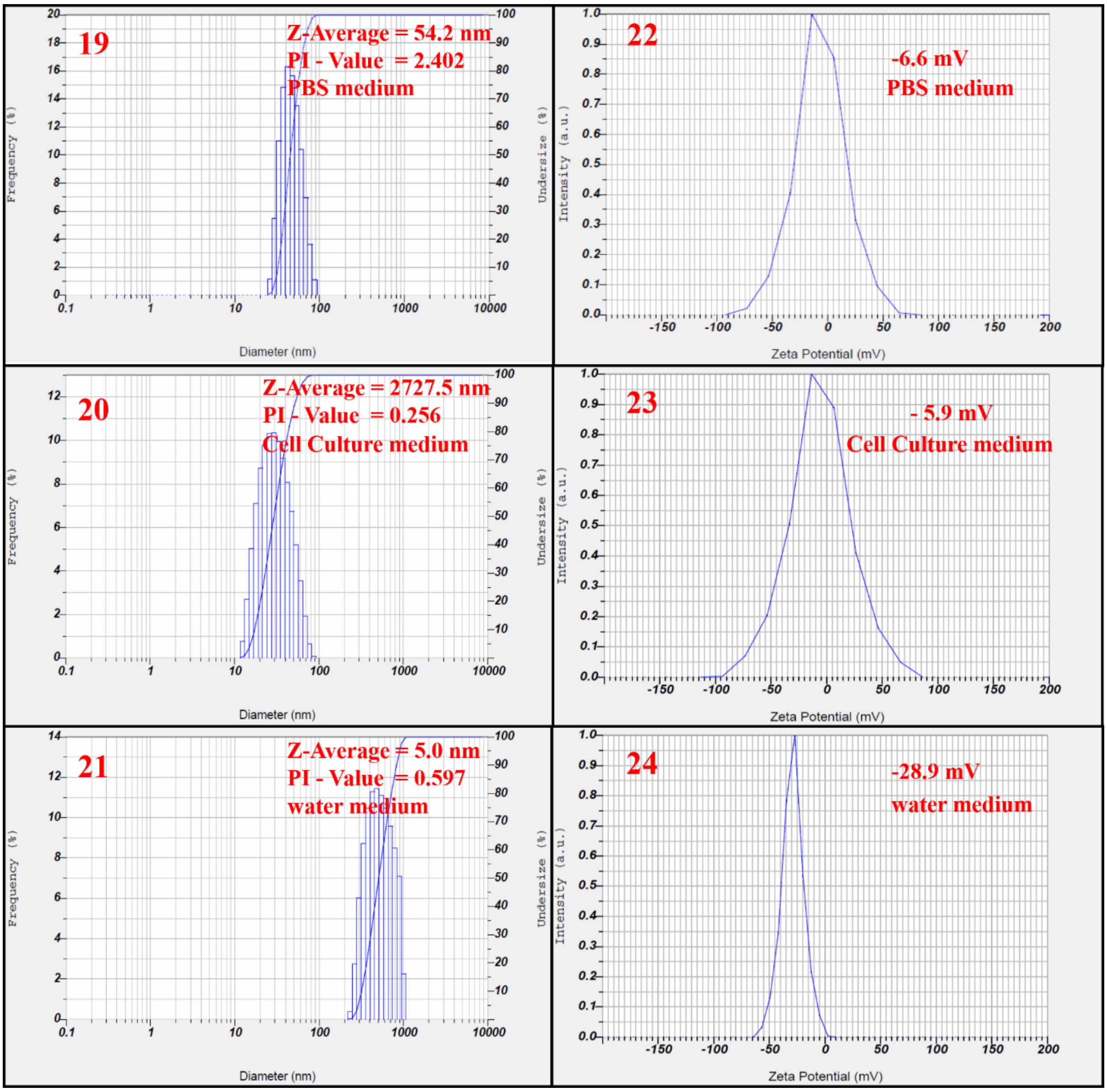

Figure S5. Hydrodynamic size distribution and Zeta potential of ZrO_2/rGO NC 0.05M (0.5 mg mL^{-1}) in PBS medium (19, 22), Cell culture medium (20, 23) and Water medium (21, 24) were measured by DLS at room temperature.

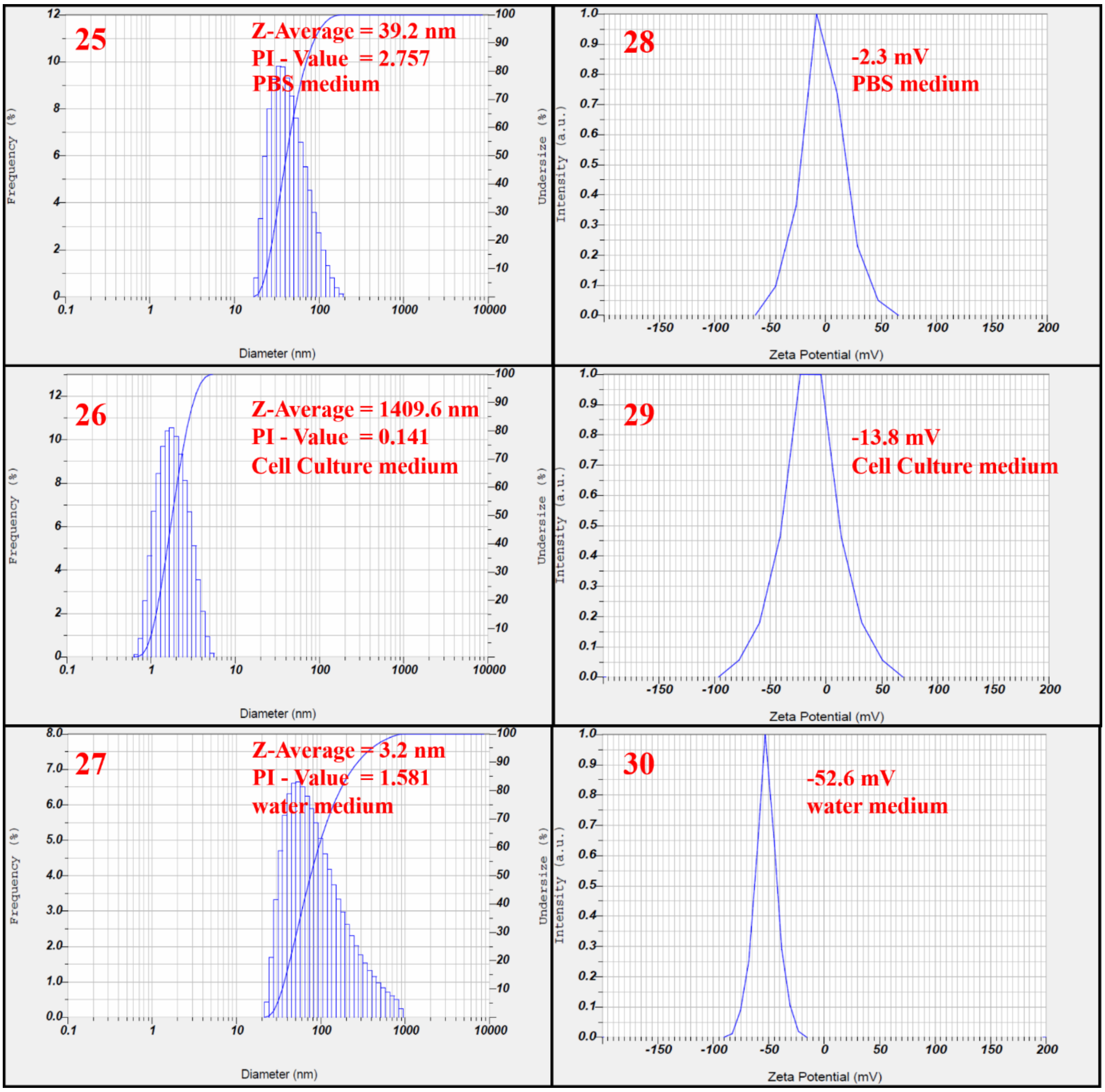

Figure S6. Hydrodynamic size distribution and Zeta potential of ZrO_2/rGO NC 0.1M (0.5 mg mL^{-1}) in PBS medium (25, 28), Cell culture medium (26, 29) and Water medium (27, 30) were measured by DLS at room temperature.

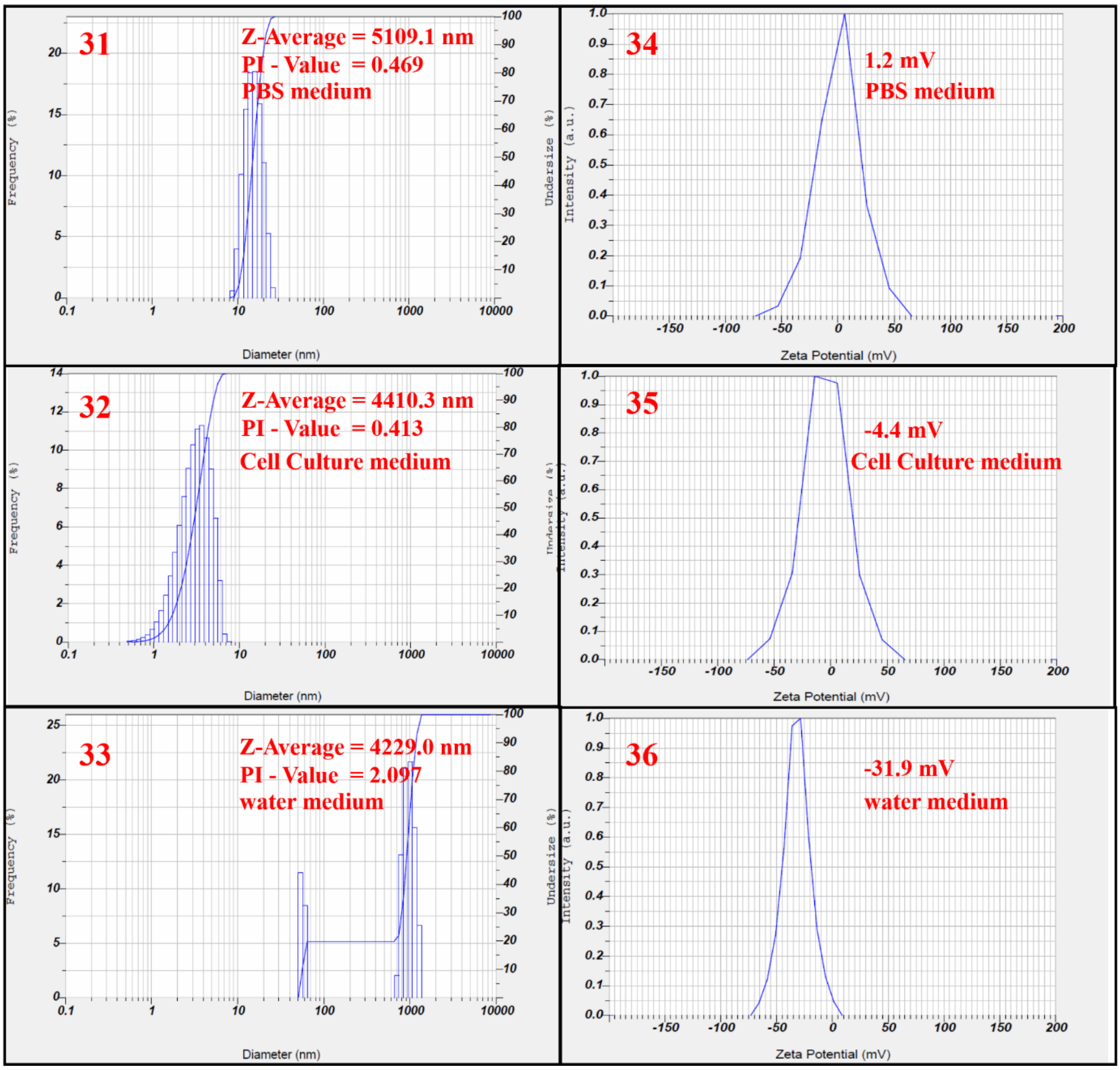

Figure S7. Hydrodynamic size distribution and Zeta potential of ZrO_2 NP 0.05M (0.5 mg mL^{-1}) in PBS medium (31, 34), Cell culture medium (32, 35) and Water medium (33, 36) were measured by DLS at room temperature.

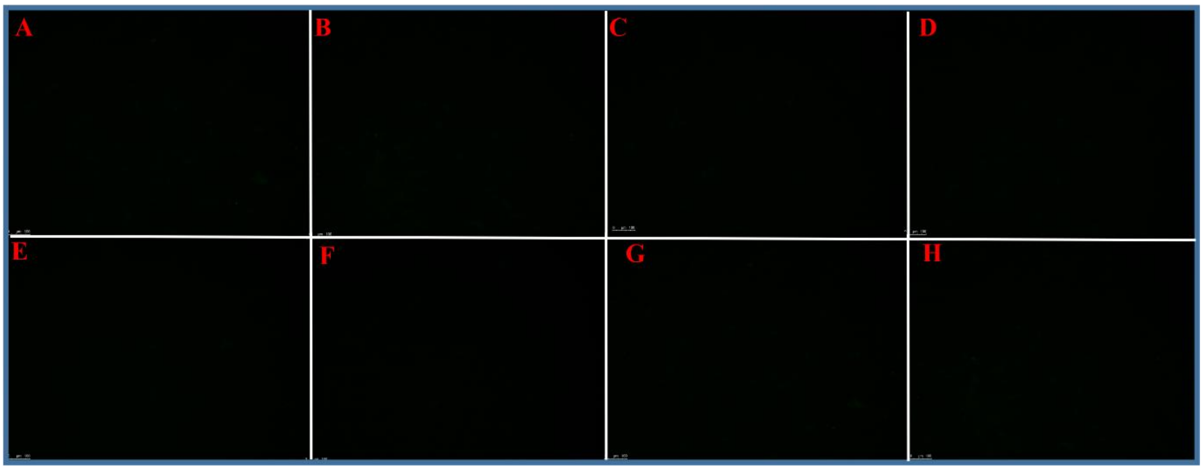

Figure S8: Representative fluorescence microscopic images of ROS generation for hMSCs normal cell line; Negative control (A), GO (B), rGO-AP (C), ZrO_2/rGO NC 0.01M (D), ZrO_2/rGO NC 0.05M (E), ZrO_2/rGO NC 0.1M (F), ZrO_2 NP 0.05M (G), Positive control drug (Cisplatin) (H). Images are representative of 3 independent experiments. Quantification of the mean fluorescence intensity is done using Image J from 3 images from each run from different groups (I). Data is average ± SE of 3 independent runs done in triplicate wells in each run (*p < 0.05).

Table S2: IC_{50} Values (µg L^{-1}) of nanomaterials GO, rGO-AP, ZrO_2/rGO NC 0.01M, ZrO_2/rGO NC 0.05M, ZrO_2/rGO NC 0.1M and ZrO_2 NP 0.05M for human cancerous cell lines.

Sample	Incubation time	A549 Cell line	HCT116 Cell line
GO	24 h	3.0215	2.7703
rGO-AP	24 h	1.7232	0.9953
ZrO_2/rGO NC 0.01M	24 h	1.3898	0.7921
ZrO_2/rGO NC 0.05M	24 h	1.4780	0.9738
ZrO_2/rGO NC 0.1M	24 h	1.3481	1.1520
ZrO_2 NP 0.05M	24 h	2.5460	2.4815

Table S3: The ROS quantified mean fluorescence intensity (MFI) data values for Negative control, GO, rGO-AP, ZrO_2/rGO NC 0.01M, ZrO_2/rGO NC 0.05M, ZrO_2/rGO NC 0.1M, ZrO_2 NP 0.05M and Positive control drug (Cisplatin) for human cancerous cell lines.

Sample	A549 Cell line (MFI) Values	HCT116 Cell line (MFI) Values
Negative control	0.39	1.81
GO	2.82	14.5
rGO-AP	3.36	14.59
ZrO_2/rGO NC 0.01M	5.65	24.53
ZrO_2/rGO NC 0.05M	4.29	18.26
ZrO_2/rGO NC 0.1M	3.94	12.34
ZrO_2 NP 0.05M	2.62	8.56
Positive control drug (Cisplatin)	3.15	9.29

Table S4: Data obtained from FITC-conjugated Annexin-V and PI staining assay for A549 cells treated with 10 μg L^{-1} each of the respective samples i.e., Negative control, Positive control drug (Cisplatin), GO, rGO-AP, ZrO_2/rGO NC 0.01M, ZrO_2/rGO NC 0.05M, ZrO_2/rGO NC 0.1M, and ZrO_2 NP 0.05M

Sample	Q1 (%)	Q2 (%)	Q3 (%)	Q4 (%)
Negative control	0	0	99.3	0.7
Positive control drug (Cisplatin)	0	22.7	1.3	76.0
GO	0.1	48.1	4.6	47.2
rGO-AP	0	16.7	4.5	78.9
ZrO_2/rGO NC 0.01M	0	41.5	0.9	57.7
ZrO_2/rGO NC 0.05M	0	42.2	1.5	56.2
ZrO_2/rGO NC 0.1M	0	25.5	2.0	72.5
ZrO_2 NPs 0.05M	0	20.7	3.2	76.1

Abbreviations: Q1: necrotic cells, Q2: late apoptotic, Q3: region denotes live cells, and Q4: apoptotic.

Table S5: Data obtained from FITC-conjugated Annexin-V and PI staining assay for HCT116 cells treated with 10 μg L^{-1} each of the respective samples i.e., Negative control, Positive

control drug (Cisplatin), GO, rGO-AP, ZrO_2/rGO NC 0.01M, ZrO_2/rGO NC 0.05M, ZrO_2/rGO NC 0.1M, and ZrO_2 NP 0.05M

Sample	Q1 (%)	Q2 (%)	Q3 (%)	Q4 (%)
Negative control	0.4	0.1	99.2	0.3
Positive control drug (Cisplatin)	0	56.0	0.4	43.6
GO	0.2	26.0	6.4	67.4
rGO-AP	0	8.4	3.9	87.7
ZrO_2/rGO NC 0.01M	0	5.6	1.5	92.8
ZrO_2/rGO NC 0.05M	0	8.2	2.1	89.7
ZrO_2/rGO NC 0.1M	0	12.4	2.1	85.6
ZrO_2 NP 0.05M	0	10.3	2.8	86.9

Abbreviations: Q1: necrotic cells, Q2: late apoptotic, Q3: region denotes live cells, and Q4: apoptotic.

Table S6A. Statistical analysis of data sets for significant evaluation of anticancer activity of ZrO_2/rGO NCs on A549 cell lines by MTT assay

	1	2	3	4	5	6
	GO	rGO-AP	ZrO2/rGO 0.01M	ZrO2/rGO 0.05M	ZrO2/rGO 0.1M	ZrO2 NP 0.05M
Concentration (ppm)	% Viability					
1	43.16	30.43	8.53	6.87	8.63	35
2	36.75	29.98	7.67	8.6	8.93	27
4	33.26	29.7	7.17	6.7	8.97	27.27
6	32.7	28.76	7.23	6.47	8.17	26.53
8	31.36	27.9	7.23	6.3	8	20.6
10	29.9	27.43	5.83	6.2	7.83	20.1
Mean	34.52	29.03	7.28	6.86	8.22	26.08
SD	4.81	1.2	8.64	0.89	0.49	5.44
Variance	23.17	1.44	74.59	0.79	0.24	29.56
Variance/n	3.8617	0.2400	12.4317	0.1317	0.0400	4.9267
Sum of variance/n		4.1017	16.2933	3.9933	3.9017	8.7883
SQRT (1 vs. others)		2.02527	4.03650	1.99832	1.97527	2.96451
t-value		2.71	6.75	13.84	13.31	2.85
df		10	10	10	10	10
critical t-value (@p=0.05)	2.23	2.23	2.23	2.23	2.23	2.23
SE/RSD	1.96	0.49	3.53	0.36	0.20	2.22
p-value (t-test)		1 vs. 2	1 vs. 3	1 vs. 4	1 vs. 5	1 vs. 6
		0.017	1.51E-05	2.29E-05	3.28E-05	0.0001
			2 vs. 3	2 vs.4	2 vs. 5	2 vs. 6
			5.54E-09	3.02E-08	1.82E-08	0.1591
				3 vs. 4	3 vs. 5	3 vs. 6
				3.21E-01	1.02E-02	0.0002
					4 vs. 5	4 vs. 6
					1.97E-03	0.0003
						5 vs. 6
						0.0004
skew (Series)	1.39	-0.30	-0.45	2.04	0.02	0.62
		Note: not skewed (skew value is				

Table S6B. Statistical analysis of data sets for significant evaluation of anticancer activity of ZrO2/rGO NCs on HCT116 cell lines by MTT assay

	1	2	3	4	5	6
	GO	rGO-AP	ZrO2/rGO 0.01M	ZrO2/rGO 0.05M	ZrO2/rGO 0.1M	ZrO2 NP 0.05M
Concentration (ppm)	% Viability					
1	39.73	26.73	24.4	25.77	27.23	38.47
2	34.07	25.43	23.93	24.73	26.13	33.37
4	33.97	24.17	22.67	24.57	25.73	32.33
6	32.83	24.47	19.43	23.57	24.7	30.27
8	31.73	23.8	17.53	19.27	21.23	28
10	29.67	22.22	14.93	18.33	20.8	27.63
Mean	33.67	24.47	20.48	22.71	24.30	31.68
SD	3.39	1.53	3.81	3.12	2.68	4.03
Variance	11.49	2.33	14.53	9.73	7.16	16.26
Variance/n	1.91	0.39	2.42	1.62	1.19	2.71
Sum of variance/n		2.30	4.34	3.54	3.11	4.63
SQRT (1 vs. others)		1.516575	2.083267	1.881489	1.763519	2.151743
t-value		6.06	6.33	5.83	5.31	0.92
df		10	10	10	10	10
critical t-value (@p=0.05)	2.23	2.23	2.23	2.23	2.23	2.23
SE/RSD	1.38	0.62	1.56	1.27	1.09	1.65
p-value (t-test)		1 vs. 2	1 vs. 3	1 vs. 4	1 vs. 5	1 vs. 6
		0.000102	1.85E-05	3.79E-05	5.28E-05	0.005992
			2 vs. 3	2 vs. 4	2 vs. 5	2 vs. 6
			1.21E-02	7.97E-02	8.00E-01	0.001236
				3 vs. 4	3 vs. 5	3 vs. 6
				7.95E-03	1.32E-03	2.35E-05
					4 vs. 5	4 vs. 6
					6.67E-04	0.000119
						5 vs. 6
						0.000261
skew (Series)	1.17	0.06	-0.47	-0.79	-0.59	0.91

Note: Not skewed (skew value is lower than 2*SQRT of 6/N)]

Table S6C: Statistical analysis of data sets for significant evaluation of anticancer activity of ZrO_2/rGO NCs on hMSCs cell lines by MTT assay

Concentration (ppm)	1	2	3	4	5	6	7	8
1	Plant extraxt	GO	rGO-AP	GO	rGO-AP	ZrO2/rGO 0.01M	ZrO2/rGO 0.05M	Cisplatin
2	% Viability	% Viability		% Viability				
4	0.228	0.206	0.206	0.191	0.181	0.21	0.226	0.127
6	0.206	0.224	0.217	0.186	0.192	0.212	0.206	0.094
8	0.202	0.212	0.217	0.171	0.199	0.217	0.215	0.062
Mean	0.212	0.214	0.2133333	0.1826667	0.1906667	0.213	0.215667	0.0943333
SD	0.014	0.00917	0.0063509	0.0104083	0.0090738	0.00360555	0.010017	0.0325013
Variance	0.000196	8.4E-05	4.033E-05	0.0001083	8.233E-05	0.000013	0.0001	0.0010563
Variance/n	6.533E-05	2.8E-05	1.344E-05	3.611E-05	2.744E-05	4.3333E-06	3.34E-05	0.0003521
Sum of variance/n	9.333E-05		0.000041	0.000064	0.000055	0.000032	6.1E-05	0.00038
SQRT (2 vs. others)	0.0096609		0.006438	0.008007	0.007446	0.005686	0.007839	0.019496
t-value	0.00050		0.00017	0.00783	0.00583	0.00025	0.00042	0.029917
df	4	4	4	4	4	4	4	4
critical t-value (@p=0.05)	2.78	2.78	2.78	2.78	2.78	2.78	2.78	2.78
SE/RSD	0.0080829	0.00529	0.0036667	0.0060093	0.0052387	0.00208167	0.005783	0.0187646
		1 vs. 2	1 vs. 3	1 vs. 4	1 vs.5	1 vs. 6	1 vs. 7	1 vs. 8
p-value (t-test)		0.44	9.20E-01	2.76E-02	2.48E-01	0.928389	0.51715	0.009595
			2 vs. 3	2 vs. 4	2 vs. 5	2 vs. 6	2 vs. 7	2 vs. 8
			0.87	0.06	0.05	0.87	0.89338	0.029811
				3 vs. 4	3 vs. 5	3 vs. 6	3 vs. 7	3 vs. 8
				0.075645	0.010431	0.909833	0.82362	0.032606
					4 vs. 5	4 vs. 6	4 vs. 7	4 vs. 8
					0.543168	0.0643385	0.04217	0.021354
						5 vs 6	5 vs 7	5 vs 8
						0.0221843	0.12996	0.056718
							6 vs. 7	6 vs. 8
							0.73154	0.029342
								7 vs. 8
								0.017514
skew (Series)	1.574344	0.9352	-1.73205	-1.29334	-0.646969	1.1520696	0.29817	0.046147

not skewed (skew value is lower than [2*SQRT(6/N) i.e. 2.8284])

References

1. Zhang, Y.; Tang, Z. R.; Fu, X.; Xu, Y. J. TiO_2−graphene nanocomposites for gas-phase photocatalytic degradation of volatile aromatic pollutant: is TiO_2−graphene truly different from other TiO_2−carbon composite materials?. *ACS Nano* **2010,** 4 (12), 7303-7314.
2. Guo, D.; Lu, Y.; Zhao, Y.; Zhang, X. Synthesis and physicochemical properties of graphene/ZrO_2 composite aerogels. *RSC Adv.* **2015,** 5(16), 11738-11744.
3. Mohan, S.; Kumar, V.; Singh, D. K.; Hasan, S. H. Synthesis and characterization of rGO/ZrO2 nanocomposite for enhanced removal of fluoride from water: kinetics, isotherm, and thermodynamic modeling and its adsorption mechanism. *RSC Adv.* 6 **2016,** (90), 87523-87538.
4. Tan, Y.; Zhu, L.; Niu, H.; Cai, Y.; Wu, F.; Zhao, X. Synthesis of flower-shaped ZrO_2–C composites for adsorptive removal of trichlorophenol from aqueous solution. *RSC Adv.* **2015,** 5 (94), 77175-77183.
5. Gurushantha, K.; Anantharaju, K. S.; Nagabhushana, H.; Sharma, S. C.; Vidya, Y. S.; Shivakumara, C.; Nagaswarupa, H. P.; Prashantha, S. C.; Anilkumar, M. R. Facile green fabrication of iron-doped cubic ZrO2 nanoparticles by Phyllanthus acidus: structural, photocatalytic and photoluminescent properties. *Journal of Molecular Catalysis A: Chemical,* **2015,** 397, 36-47.
6. Xu, R. Particle Characterization: Light Scattering Methods, Springer Science & Business Media, **2001**.
